# Islet‐Resident Memory T Cells Orchestrate the Immunopathogenesis of Type 1 Diabetes through the FABP4‐CXCL10 Axis

**DOI:** 10.1002/advs.202308461

**Published:** 2024-06-17

**Authors:** Xiaoping Wu, Lai Yee Cheong, Lufengzi Yuan, Leigang Jin, Zixuan Zhang, Yang Xiao, Zhiguang Zhou, Aimin Xu, Ruby LC Hoo, Lingling Shu

**Affiliations:** ^1^ State Key Laboratory of Pharmaceutical Biotechnology The University of Hong Kong Hong Kong 999077 P. R. China; ^2^ Department of Pharmacology & Pharmacy The University of Hong Kong Hong Kong 999077 P. R. China; ^3^ Department of Medicine The University of Hong Kong Hong Kong 999077 P. R. China; ^4^ Second Xiangya Hospital Key Laboratory of Diabetes Immunology National Clinical Research Center for Metabolic Diseases Central South University Changsha Hunan 410083 P. R. China; ^5^ State Key Laboratory of Oncology in South China Guangdong Provincial Clinical Research Center for Cancer, Department of Hematological Oncology Sun Yat‐sen University Cancer Center Guangzhou 510060 P. R. China

**Keywords:** CXCL10, CXCR3, FABP4, tissue‐resident memory T cells, Type 1 diabetes

## Abstract

Type 1 diabetes (T1D) is a chronic disease characterized by self‐destruction of insulin‐producing pancreatic β cells by cytotoxic T cell activity. However, the pathogenic mechanism of T cell infiltration remains obscure. Recently, tissue‐resident memory T (*T*_RM_) cells have been shown to contribute to cytotoxic T cell recruitment. *T*_RM_ cells are found present in human pancreas and are suggested to modulate immune homeostasis. Here, the role of *T*_RM_ cells in the development of T1D is investigated. The presence of *T*_RM_ cells in pancreatic islets is observed in non‐obese diabetic (NOD) mice before T1D onset. Mechanistically, elevated fatty acid‐binding protein 4 (FABP4) potentiates the survival and alarming function of *T*_RM_ cells by promoting fatty acid utilization and C‐X‐C motif chemokine 10 (CXCL10) secretion, respectively. In NOD mice, genetic deletion of FABP4 or depletion of *T*_RM_ cells using CD69 neutralizing antibodies resulted in a similar reduction of pancreatic cytotoxic T cell recruitment, a delay in diabetic incidence, and a suppression of CXCL10 production. Thus, targeting FABP4 may represent a promising therapeutic strategy for T1D.

## Graphical abstract

During the development of Type 1 diabetes, increased accumulation of tissue‐resident memory T (*T*_RM_) cells in pancreatic islets exert detrimental effects by sending alarming signals to recruit cytotoxic T cells thus causing β cell destruction. Mechanistically, elevated FABP4 potentiates the survival and alarming function of *T*_RM_ cells by promoting fatty acid utilization and a chemokine CXCL10 secretion, respectively.

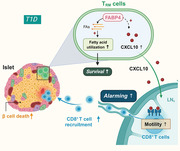

## 1 Introduction

Type 1 diabetes (T1D), also known as autoimmune diabetes, is an organ‐specific autoimmune disease precipitated by immune‐associated destruction of insulin‐producing pancreatic β cells.^[^ ^1^ ^]^ T1D can lead to hyperglycemia‐related microvascular complications and cardiovascular diseases, which primarily account for the shorter life expectancy among T1D patients.^[^ ^2^ ^]^ However, there is currently no cure available for T1D. Most of the patients have to rely on lifelong insulin injections. Although administration of insulin transformed T1D into a potentially treatable disease, it also triggers life‐threatening complications such as hypoglycemia and ketoacidosis.^[^ ^2^ ^]^ Manipulating the immune attack on β cells has emerged as a promising intervention for T1D in recent years.^[^ ^3^ ^]^ Therefore, understanding the complex immune cell interaction within the pancreatic islets is vital for developing effective therapies to manage T1D.

Adaptive immunity plays a critical role in the progression of T1D.^[^ ^3^ ^]^ Among various types of T cells, cytotoxic CD8^+^ T cells are predominantly responsible for β cell destruction. Upon activation, CD8^+^ T cells recognize major histocompatibility complex (MHC) class I molecules expressed on β cells, triggering the secretion of cell death‐inducing cytokines such as interferon‐gamma (IFNγ) and membrane‐disrupting proteins perforin and granzyme B thus promoting β cell apoptosis. Interaction with other immune cells also enhances the activation state of CD8^+^ T cells. CD4^+^ T cells activate macrophages and dendritic cells, which facilitate the maturation and activation of CD8^+^ T cells.^[^ ^4^ ^]^ Tissue‐resident memory T cells (*T*_RM_) were recently identified in human pancreas and were suggested to play a critical role in maintaining immune homeostasis^[^ ^5^, ^6^ ^]^ while in patients with pancreatitis, the immune tolerance of *T*_RM_ cells regulated by PD1/PD‐L1 pathway is impaired.^[^ ^6^ ^]^ CD8^+^CD69^+^CD103^+^ *T*_RM_ cells, which contribute to 43% of the CD8^+^ T cell population in insulitis lesions of patients with recent‐onset T1D, display significantly different inflammatory profile from that of the classic CD8^+^ T cells as assessed.^[^ ^5^ ^]^ CD69 is essential to *T*_RM_ cell retention preventing their egress into the circulation^[^ ^7^ ^]^ while CD103 is an adhesion molecule involved in initial accumulation.^[^ ^8^ ^]^ *T*_RM_ cells are identified as a subset of memory T cells that exhibit long‐term persistency in peripheral tissue in the absence of antigens. Under physiological conditions, *T*_RM_ cells provide rapid local immune protection against the reinfection of pathogens.^[^ ^9^ ^]^ Nevertheless, immunological memory is a double‐edged sword in the immune system. When exposed to autoantigens, pathogenic *T*_RM_ cells are activated and induce autoimmune attacks.^[^ ^9^ ^]^ On one hand, *T*_RM_ cells release inflammatory chemokines to recruit circulating T cells from the peripheral blood, which is known for its alarming function.^[^ ^10^, ^11^ ^]^ On the other hand, *T*_RM_ cells lyse the infected targets directly by expressing granzyme B.^[^ ^10^ ^]^ *T*_RM_ cells have been shown to play key roles in various autoimmune diseases such as mycosis fungoides and psoriasis.^[^ ^9^ ^]^ These findings implicating *T*_RM_ cells may be involved in the development of T1D.

In the present study, by using non‐obese diabetic (NOD) mice and neutralizing antibody‐mediated *T*_RM_ cell depletion, we demonstrated that *T*_RM_ cells play a pivotal role in modulating cytotoxic T cell recruitment thus promoting the development of T1D. Mechanistically, elevated fatty acid‐binding protein 4 (FABP4) upregulates the fatty acid oxidation thus prolonging the *T*_RM_ cell survival in the pancreatic islets. In addition, FABP4 promotes CXCL10 production from *T*_RM_ cells, which potentiates *T*_RM_ cells to recruit CD8^+^ T cells into the pancreas. Taken together, our results identified *T*_RM_ cells as critical mediators in adaptive immune activation in T1D. The mechanistic study sheds light on FABP4 which might be a potential therapeutic target for autoimmune diabetes.

## 2 Results

### 2.1 Pancreatic *T*_RM_ Cell Recruitment During T1D Progression is FABP4‐Dependent

**Figure 1:**
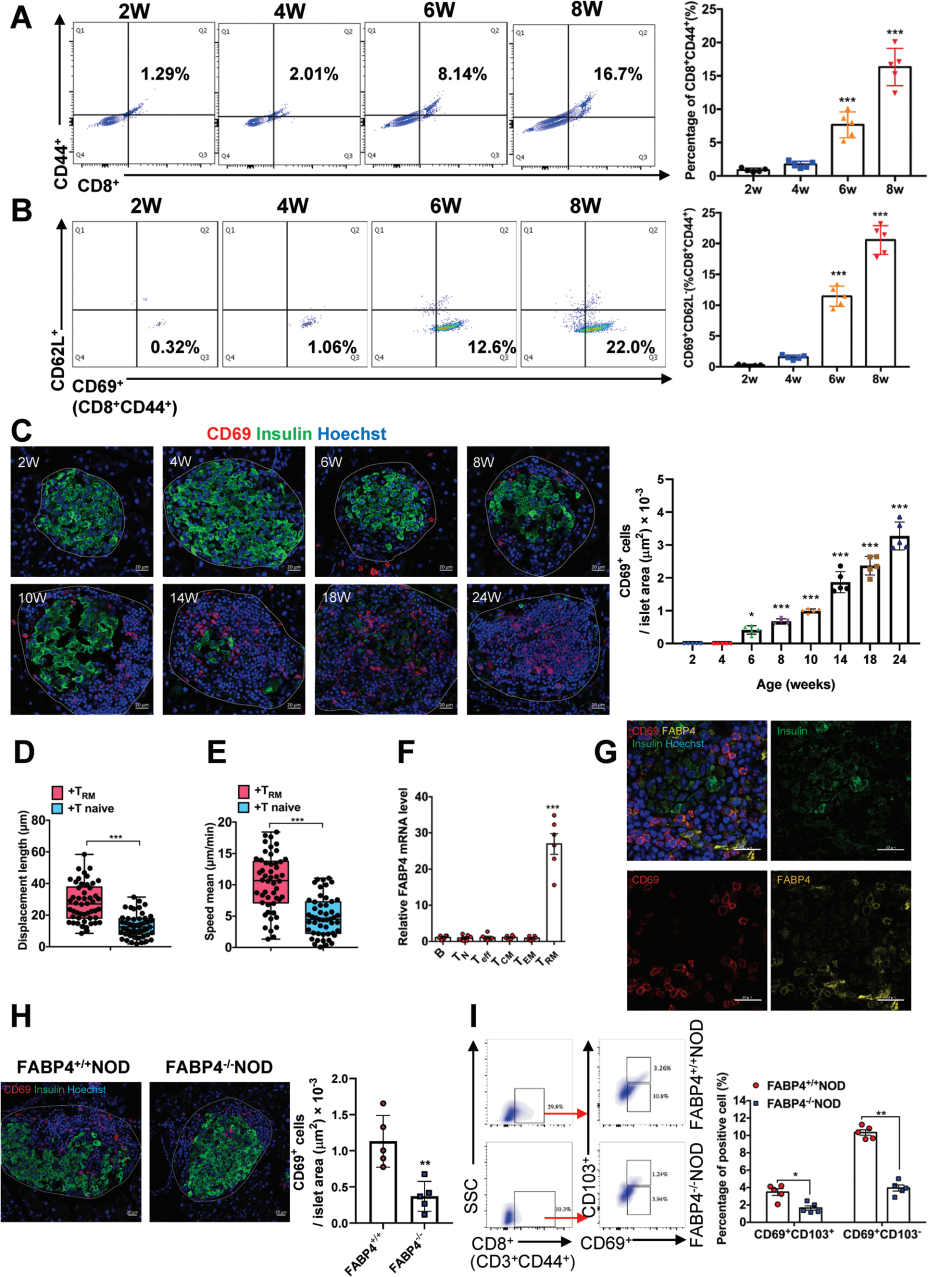
Pancreatic *T*_RM_ cell recruitment during T1D progression is FABP4‐dependent. A) Infiltration of antigen‐experienced CD8^+^ T cells (CD3^+^CD8^+^CD44^+^) in pancreatic islets of NOD mice at 2, 4, 6, and 8 weeks old (2 to 8 W). The right panel is the quantification (*n* = 5). B) Infiltration of tissue‐resident memory T cells (CD3^+^CD8^+^CD44^+^CD69^+^CD62L^−^) in pancreatic islets of NOD mice at 2, 4, 6, and 8 weeks old (2 to 8 W). The right panel is the quantification (*n* = 5). C) Representative images of immunofluorescent co‐staining of CD69 and insulin in the pancreas of NOD mice at different ages as indicated (Scale bar 20 µm). The right panel is the quantification of the number of CD69^+^ cells within the islet area (*n* = 5). D,E) Quantification of (D) displacement length and (E) average speed analyzed by Imaris showing the motility of GFP‐labeled CD8^+^ T cells cultured in the conditioned medium of *T*_RM_ or naïve T cells for 24 h. F) The mRNA abundance of FABP4 in isolated B cells (B), naive T cells (*T*_N_), effector T cells (*T*_eff_), central memory T cells (*T*_CM_), effector memory T cells (*T*_EM_), and *T*_RM_ cells from the pancreas of 10‐week‐old NOD mice (*n* = 6). G) Representative images of immunofluorescent co‐staining of FABP4, CD69, and insulin in the pancreas of 10‐week‐old NOD mice (Scale bar 20 µm). H) Representative images of immunofluorescent co‐staining of CD69 and insulin in the pancreas of 10‐week‐old FABP4^+/+^NOD and FABP4^−/−^NOD mice (Scale bar 20 µm). The right panel is the quantification of the number of CD69^+^ cells within the islet area (*n* = 5). I) Representative FACS plots showing the abundance of *T*_RM_ cells in the pancreas of 10‐week‐old FABP4^+/+^NOD and FABP4^−/−^NOD mice. The right panel is the quantification (*n* = 5). Data are expressed as mean ± SD. ^*^ *p* < 0.01, ^**^ *p* < 0.01, ^***^ *p* < 0.001. Unpaired Student's *t* test or Mann‐Whitney *U* test was used in (D), (E), and (H). ANOVA followed by Sidak's test was used in (A), (B), (C), (F), and (I).

During the spontaneous T1D progression in NOD mice starting from the age of 6 weeks old, there was a significant increase in the infiltration of cytotoxic CD8^+^ T cells (CD44^+^CD8^+^) and *T*_RM_ cells (CD44^+^CD8^+^CD62L^−^CD69^+^) in the mouse pancreas (**Figure** **1**A,B). Consistently, the gradual accumulation of CD69^+^ *T*_RM_ cells surrounding pancreatic islets was further confirmed in NOD mice from 6 weeks old to later stages by immunofluorescent staining (Figure 1C). *T*_RM_ cells are capable of recruiting other immune cells to the injury site by releasing chemokines or cytokines known as *T*_RM_ cell alarming.^[^ ^10^ ^]^ Next, CD8^+^ T cells were cultured in the conditioned medium of either pancreatic *T*_RM_ or naive T cells to compare their alarming function. The motility of CD8^+^ T cells, indicating the movement of T cells searching for signaling partners or targets,^[^ ^12^ ^]^ cultured in the conditioned medium of *T*_RM_ cell was significantly upregulated as indicated by the enhanced track displacement length and track speed (Figure 1D,E). The early presence of *T*_RM_ cells in the pancreas and its regulation of CD8^+^ T cell motility implicated that pancreatic *T*_RM_ cells may play an essential role in activating adaptive immunity during T1D progression.

Since pancreatic *T*_RM_ cells maintain immune homeostasis under normal conditions^[^ ^6^ ^]^ while they accumulate in insulitis lesions of patients with recent‐onset T1D^[^ ^5^ ^]^ and proposed to trigger an adaptive immune attack in the present study, we next explored the key mediators modulating the transition of *T*_RM_ cells from “immunoprotective” to “immunoaggressive” state. Fatty acid‐binding protein 4 (FABP4) is known to maintain the longevity and inflammatory function of *T*_RM_ cells in human and rodent skin.^[^ ^13^ ^]^ It is also a potential biomarker for pre‐eclampsia prediction in women with T1D^[^ ^14^ ^]^ and we have shown that macrophage‐derived FABP4 enhances CD8^+^ T cell activation and shift of CD4^+^ helper T cells toward Th1 subtypes during the onset of T1D.^[^ ^15^ ^]^ We hypothesized that FABP4 exacerbates adaptive immune activation through regulating *T*_RM_ cell function.

Among various adaptive immune cells isolated from the pancreatic islets of 10‐week‐old NOD mice, *T*_RM_ cells exhibited the highest FABP4 mRNA abundance (Figure 1F; Figure S1A, Supporting Information). The expression of FABP4 in *T*_RM_ cells surrounding β cells was evidenced as indicated by the co‐localization of FABP4 and CD69^+^ cells in the pancreas of 10‐week‐old NOD mice (Figure 1G). Furthermore, the presence of *T*_RM_ cells in the pancreas of FABP4^−/−^ NOD mice was significantly reduced when compared to that of FABP4^+/+^ NOD mice (Figure 1H). In addition to the tissue retention marker CD69,^[^ ^7^ ^]^ *T*_RM_ cells include CD103^+^ and CD103^−^ subsets.^[^ ^11^ ^]^ CD103^+^ *T*_RM_ cells exhibit higher cytotoxicity expressing more granzymes, while CD103^−^ *T*_RM_ cells are more proliferative, exhibit higher sensitivity to T cell receptor stimulation, and produce more cytokines and chemokines.^[^ ^11^ ^]^ In 10‐week‐old NOD mouse pancreas, the percentage of CD103^−^ *T*_RM_ cells was higher than that of CD103^+^ *T*_RM_ cells implicating that the proliferative CD103^−^ *T*_RM_ cells contribute to the increased *T*_RM_ cell abundance. Notably, the populations of both CD103^+^ *T*_RM_ cells and CD103^−^ *T*_RM_ cells were significantly lower in the pancreas of FABP4^−/−^ NOD mice compared to that of FABP4^+/+^ NOD mice (Figure 1I) while there was no difference in the population of T_CM_ cells and T_EM_ cells (Figure S1B, Supporting Information). This data implicated that FABP4 generally potentiates pancreatic *T*_RM_ cell abundance without a discrepancy in modulating CD103^+^ and CD103^−^ *T*_RM_ cell subsets.

Collectively, these findings suggested that FABP4 is a key mediator potentiating *T*_RM_ cell accumulation in the pancreas during the progression of T1D. It is also of great importance to identify the continuous increase of *T*_RM_ cells in islets before diabetic onset which implicates the pathogenic effect of *T*_RM_ cells in the initiation stage of an autoimmune attack.

### 2.2 FABP4 Promotes *T*_RM_ Cell Survival in the Pancreas of NOD Mice

**Figure 2:**
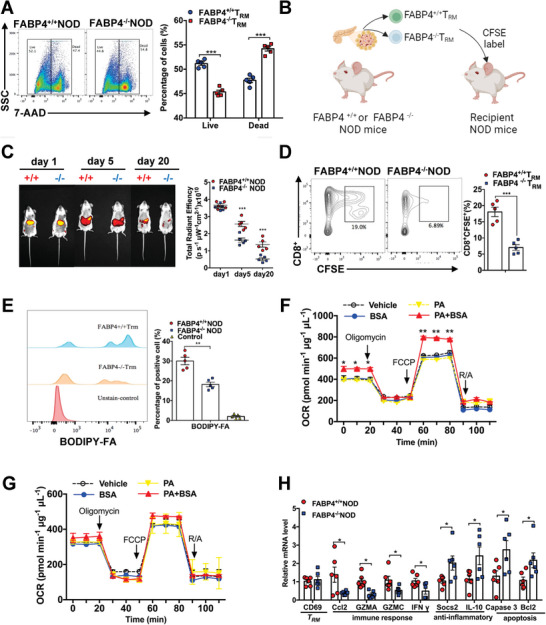
FABP4 promotes the survival of *T*_RM_ cells in the pancreas of NOD mice. A) Representative FACS plots showing the spontaneous apoptotic rate of *T*_RM_ cells. The right panel is the quantification of the percentage of 7‐ADD^+^ apoptotic cells (*n* = 5). B) Schematic diagram showing the carboxyfluorescein succinimidyl ester (CFSE)‐labeled FABP4^+/+^ *T*_RM_ or FABP4^−/−^ *T*_RM_ cells isolated from NOD mouse pancreatic islet was transferred to FABP4^+/+^ NOD mice (i.v., 3 × 10^6^ cells/mice). C) Representative images showing the fluorescence of CFSE‐positive cells in recipient mice from day 1 to day 20 using the IVIS system. The right panel is the quantification of fluorescent intensity (*n* = 5). D) Representative FACS plots showing the CFSE‐positive CD8^+^ T cells in the pancreas of NOD mice received FABP4^+/+^ or FABP4^−/−^ *T*_RM_ cell after 20 days (*n* = 5). E) Representative FACS plots showing the uptake of BODIPY‐labeled fatty acid (BODIPY‐FA) in FABP4^+/+^ *T*_RM_ and FABP4^−/−^ *T*_RM_ cells. The right panel is the quantification of cells with a BODIPY positive signal (*n* = 5). F,G) Oxygen consumption rate (OCR) of (F) FABP4^+/+^ *T*_RM_ and (G) FABP4^−/−^ *T*_RM_ cells treated with palmitic acid (PA, 200 nm) with or without pre‐incubation with BSA (0.3%) (*n* = 3). OCR, oxygen consumption rate; FCCP, carbonyl cyanide‐4‐(trifluoromethoxy) phenylhydrazone; R/A, rotenone/antimycin A. H) The mRNA abundance of gene markers related to immune response, anti‐inflammation, and apoptosis of *T*_RM_ cells isolated from FABP4^+/+^ and FABP4^−/−^ NOD mice (*n* = 6). Data are expressed as mean ± SD. ^*^ *p* < 0.05, ^**^ *p *< 0.01, ^***^ *p* < 0.001. Unpaired Student's *t* test or Mann‐Whitney *U* test was used in (A), (C), (D), (F), and (H). ANOVA followed by Sidak's test was used in (E).

Since FABP4^+/+^ *T*_RM_ and FABP4^−/−^ *T*_RM_ cells exhibited similar proliferative rates (Figure S1C, Supporting Information), we next investigated whether FABP4 regulates *T*_RM_ cell survival. FABP4^+/+^ *T*_RM_ and FABP4^−/−^ *T*_RM_ cells isolated from the pancreas of NOD mice were cultured in the conditioned medium to determine their spontaneous apoptosis. The apoptotic rate of FABP4^−/−^ *T*_RM_ cells was significantly higher than that of FABP4^+/+^ *T*_RM_ (**Figure** **2**A). To determine whether FABP4 promotes *T*_RM_ cell survival in mice, freshly isolated FABP4^+/+^ *T*_RM_ and FABP4^−/−^ *T*_RM_ cells were labeled with carboxyfluorescein succinimidyl ester (CFSE) and adoptively transferred into recipient NOD mice^[^ ^16^ ^]^ (Figure 2B). The dynamic fluorescence of CFSE showed the presence of transplanted FABP4^+/+^ *T*_RM_ cells in the viscera of recipient NOD mice for up to 20 days while the abundance of FABP4^−/−^ *T*_RM_ cells was barely detected at day 20 (Figure 2C). Furthermore, the population of transferred FABP4^−/−^CD8^+^CFSE^+^ T cells infiltrated in the pancreas of recipient mice was significantly lower (≈3 folds) than that of FABP4^+/+^CD8^+^CFSE^+^ T cells (Figure 2D). This evidence implicated that FABP4 prolongs the survival of *T*_RM_ cells in the pancreas of NOD mice.

FABP4 and FABP5 modulated exogenous lipid uptake and mitochondrial fatty acid oxidation (FAO) are necessary for the survival of skin *T*_RM_ cells.^[^ ^13^ ^]^ Our data further demonstrated that pancreatic FABP4^−/−^ *T*_RM_ cells displayed an impaired fatty acid uptake capacity as indicated by the reduced positive cells with BIODIPY‐labelled fatty acid compared to that of FABP4^+/+^ *T*_RM_ cells (Figure 2E). Moreover, compared to FABP4^+/+^ *T*_RM_ cells, FABP4 deficiency impaired fatty acid‐stimulated mitochondrial oxidative phosphorylation in *T*_RM_ cells as indicated by the basal and FCCP‐induced maximum oxygen consumption rate (Figure 2F,G). Notably, the gene expression of apoptotic markers (caspase 3 and Bcl2) was significantly higher in FABP4^−/−^ *T*_RM_ cells when compared to those of FABP4^+/+^ *T*_RM_ cells isolated from mouse pancreas (Figure 2H). Since mitochondrial FAO plays a determinant role in *T*_RM_ cell survival,^[^ ^17^ ^]^ these findings indicated that upregulated FABP4 in *T*_RM_ cells enhances fatty acid uptake and promotes mitochondrial FAO activity, thus prolonging the *T*_RM_ cell survival. Furthermore, given that *T*_RM_ cells can gain effector‐like properties and release pro‐inflammatory cytokines^[^ ^18^ ^]^ and FABP4 overexpression potentiates inflammatory response in various cell types such as macrophages,^[^ ^19^ ^]^ whether FABP4 modulates the inflammatory response in *T*_RM_ cells was determined. The mRNA abundance of proinflammatory cytokines (CCL2 and IFNγ)^[^ ^20^ ^]^ and granzymes (GZMA and GZMC)^[^ ^21^, ^22^ ^]^ with potential β cell toxicity was significantly higher in FABP4^+/+^ *T*_RM_ cells than that of FABP4^−/−^ *T*_RM_ cells, while the expression of anti‐inflammatory genes (Socs2, IL‐10) was higher in FABP4^−/−^ *T*_RM_ cells (Figure 2H). Collectively, these data indicated that FABP4 participates in adaptive immunity in NOD mouse pancreas by potentiating *T*_RM_ cell survival through promoting fatty acid utilization and potentially inducing pro‐inflammatory response.

### 2.3 FABP4 Potentiates the Alarming Function of *T*_RM_ Cells in the Pancreatic Islets of NOD Mice

**Figure 3:**
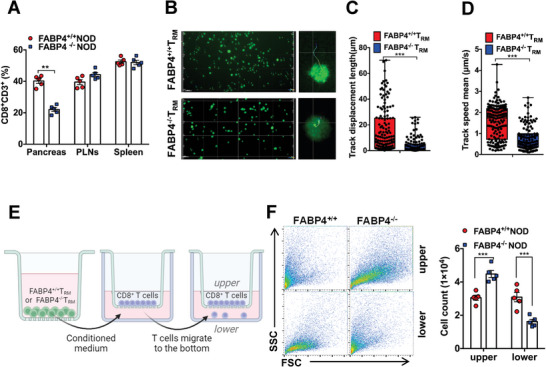
FABP4 potentiates the alarming function of *T*_RM_ cells in the pancreatic islets of NOD mice. A) Percentage of CD8^+^CD3^+^ T cells in the pancreas, pancreatic lymph nodes (PLNs), and spleen of 10‐week‐old FABP4^+/+^ NOD and FABP4^−/−^ NOD mice (*n* = 5). B–D) Representative images (B) and quantification of displacement length (C) and average speed (D) showing the motility of GFP‐labeled CD8^+^ T cells cultured in a conditioned medium of FABP4^+/+^ *T*_RM_ or FABP4^−/−^ *T*_RM_ cells analyzed by Imaris (scale bar 50 µm). E) Diagram showing the protocol for CD8^+^ T cells migration assay. F) The representative FACS plots and quantification showing the resident and migrated CD8^+^ T cells cultured in a Trans‐well plate with the conditioned medium collected from FABP4^+/+^ *T*_RM_ cells or FABP4^−/−^ *T*_RM_ cells (*n* = 5). Data are expressed as mean ± SD. ^**^ *p* < 0.01, ^***^ *p* < 0.001. Unpaired Student's *t* test or Mann‐Whitney *U* test was used in (A), (C), (D), and (F).

*T*_RM_ cells are responsible for immunological memory, serving as local sensors for previously encountered antigens and releasing innate‐like alarm signals, which stimulate the migration of cytotoxic CD8^+^ T cells into the infected pancreatic tissue.^[^ ^10^ ^]^ We next determined whether FABP4 is involved in regulating the alarming function of *T*_RM_ cells. The number of infiltrated CD8^+^ T cells in pancreas was significantly lower in FABP4^−/−^ NOD mice than in FABP4^+/+^ NOD mice, while there was no significant difference in the abundance of CD8^+^ T cells in pancreatic lymph nodes (PLNs) and spleen between the two groups (**Figure** **3**A) indicating that the recruitment of CD8^+^ T cells into pancreas is attenuated in FABP4^−/−^ NOD mice. To assess the role of FABP4 in *T*_RM_ cell‐mediated CD8^+^ T cell recruitment, primary CD8^+^ T cells were cultured in the conditioned medium of FABP4^+/+^ *T*_RM_ and FABP4^−/−^ *T*_RM_ cells. The motility of CD8^+^ T cells cultured in the conditioned medium of FABP4^+/+^ *T*_RM_ cells was significantly higher than those cultured in the conditioned medium of FABP4^−/−^ *T*_RM_ cells as evaluated by the track displacement length (15.33 µm ± 1.42 µm VS 4.20 µm ± 0.54 µm,) and track speed (1.54 µm s^−1^ ± 0.07 µm s^−1^ VS 0.79 µm s^−1^ ± 0.05 µm s^−1^) (Figure 3B–D). To confirm whether the migration activity of CD8^+^ T cells is enhanced under the stimulation of FABP4^+/+^ *T*_RM_ cells, the CD8^+^ T cells were cultured in the insert of a trans‐well plate with a conditioned medium of FABP4^+/+^ *T*_RM_ or FABP4^−/−^ *T*_RM_ cells. The number of CD8^+^ T cells present on the lower surface indicates the migration ability (Figure 3E). Consistently, the number of migrated CD8^+^ T cells cultured in the conditioned medium of FABP4^+/+^ *T*_RM_ was significantly higher than that cultured in the conditioned medium of FABP4^−/−^ *T*_RM_ cells (Figure 3F). The evidence suggests that FABP4 potentiates the immunological memory of *T*_RM_ cells by enhancing its alarming function, which promotes the recruitment of cytotoxic T cells.

### 2.4 *T*_RM_ Cells Enhance the Immunopathologic Development of T1D Through FABP4‐CXCL10 Axis

**Figure 4:**
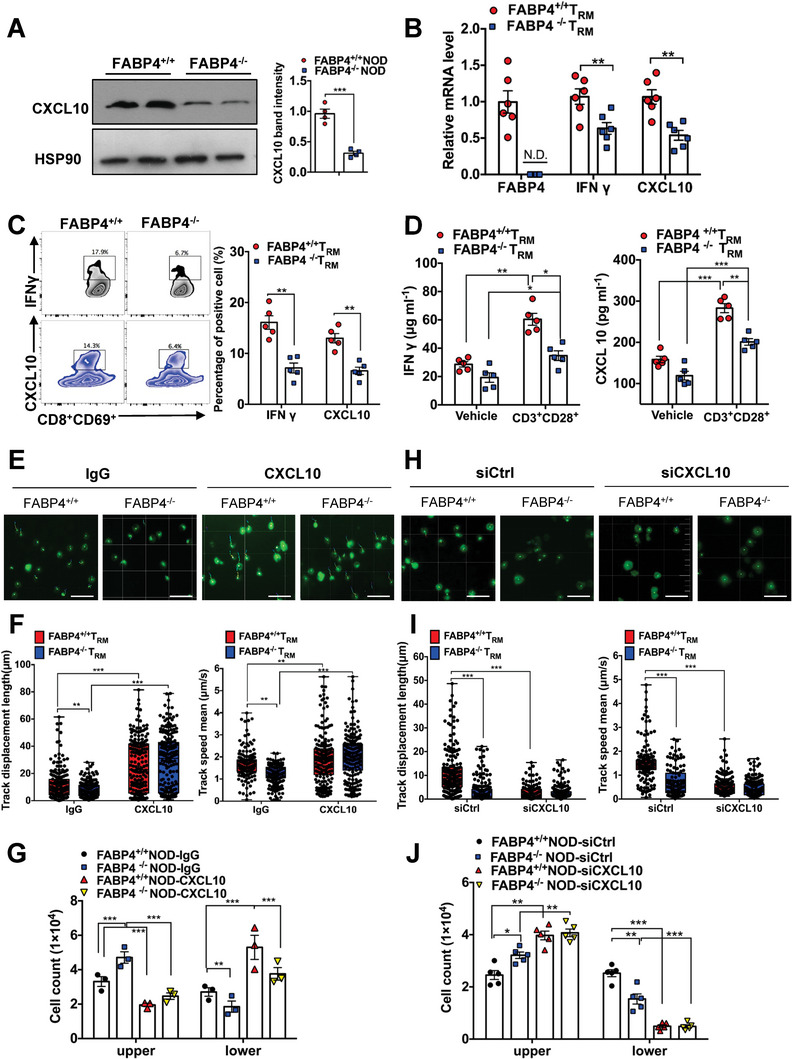
*T*_RM_ cells enhance the immunopathologic development of T1D through the FABP4‐CXCL10 axis. A) Representative immunoblots of CXCL10 and HSP90 in the pancreas of FABP4^+/+^ NOD and FABP4^−/−^ NOD mice. The right panel is the quantification of the band intensity of CXCL10 relative to HSP90 (*n* = 4). B) The mRNA abundance of FABP4, IFNγ, and CXCL10 in FABP4^+/+^ *T*_RM_ cells and FABP4^−/−^ *T*_RM_ cells isolated from NOD mice (*n* = 6). C) Representative FACS plots showing the abundance of IFNγ and CXCL10 in FABP4^+/+^ *T*_RM_ cells or FABP4^−/−^ *T*_RM_ cells. The right panel is the quantification of positive cell percentage (*n* = 5). D) Concentration of IFNγ and CXCL10 in the medium of FABP4^+/+^ *T*_RM_ cells or FABP4^−/−^ *T*_RM_ cells stimulated with CD3^+^CD28^+^ beads or vehicle (*n* = 6). E,F) Representative images (E) and quantification (F) of displacement length (left) and average speed (right) analyzed by Imaris showing the motility of GFP‐labeled CD8^+^ T cells cultured in the conditioned media of FABP4^+/+^ *T*_RM_ or FABP4^−/−^ *T*_RM_ cells in the presence or absence of recombinant CXCL10 protein (2 µg mL^−1^) or IgG control (2 µg mL^−1^) for 24 h (scale bar 50 µm). G) The number of residual (upper) and migrated (lower) CD8^+^ T cells cultured in the trans‐well plate with a conditioned medium of FABP4^+/+^ *T*_RM_ cells or FABP4^−/−^ *T*_RM_ cells in the presence or absence of recombinant CXCL10 protein (2 µg mL^−1^) or IgG (2 µg mL^−1^) for 24 h (*n* = 3). H,I) Representative pictures (H) and quantification (I) of displacement length (left) and average speed (right) showing the motility of GFP‐labeled CD8^+^ T cells cultured in the conditioned medium of FABP4^+/+^ *T*_RM_ or FABP4^−/−^ *T*_RM_ cells treated with siCXCL10 (5 nmol) or siControl (siCtrl, 5 nmol) for 24 h (scale bar 50 µm). J) The number of residual (upper) and migrated (lower) CD8^+^ T cells cultured in the trans‐well plate with the conditioned medium from FABP4^+/+^ *T*_RM_ cells or FABP4^−/−^ *T*_RM_ cells treated with siCXCL10 or siCtrl (5 nmol) for 24 h (*n* = 5). Data are expressed as mean ± SD. ^*^ *p* < 0.05, ^**^ *p* < 0.01, ^***^ *p *< 0.001. Unpaired Student's *t* test or Mann‐Whitney *U* test was used in (A), (B), and (C). ANOVA followed by Sidak's test was used in (D), (F), (G), (I), and (J).

Since CD8^+^ T cells are recruited into infected tissues mainly triggered by CXC family chemokines including C‐X‐C motif chemokine (CXCL) −9 and −10 stimulation on their surface C‐X‐C motif chemokine receptor 3 (CXCR3),^[^ ^23^ ^]^ and CXCL10 was originally identified as an IFNγ inducible protein in T1D,^[^ ^24^ ^]^ we next investigated whether FABP4 promotes recruitment of CD8^+^ T cells into pancreatic islets via enhancing the secretion of CXCL10 from *T*_RM_ cells. Analysis of transcriptional datasets from NOD mice revealed an increase in pancreatic CXCL10 and CXCR3 expression during progression of the disease (Figure S2A, Supporting Information). The induction of CXCL10 mRNA expression was significantly higher in FABP4^+/+^ NOD mice starting from 8 weeks old when compared to that of FABP4^−/−^ NOD mice, while the expression of CXCL9 was comparable between the two groups (Figure S2B, Supporting Information). In the mouse pancreas, the protein abundance of CXCL10 was significantly reduced in FABP4^−/−^ NOD mice (**Figure** **4**A). Moreover, both mRNA and protein expression of IFNγ and CXCL10 in *T*_RM_ cells from FABP4^−/−^NOD mice were significantly attenuated compared to that of FABP4^+/+^ NOD mice (Figure 4B,C). Consistently, the induction of IFNγ and CXCL10 in FABP4‐deficient *T*_RM_ cells upon stimulation of CD3^+^CD28^+^ beads, which induces in vitro activation of T cells without antigen‐presenting cells,^[^ ^25^ ^]^ was also attenuated (Figure 4D). The concentrations of CXCL10 and IFNγ in the pancreas of both 8‐week‐old and 20‐week‐old FABP4^−/−^ NOD mice were also significantly reduced compared to respective FABP4^+/+^ NOD mice (Figure S2C, Supporting Information). Since β cell is one of the major sources of CXCL10 in the insulitis lesion of NOD mice,^[^ ^26^ ^]^ the mRNA abundance and concentration of CXCL10 in β cells and *T*_RM_ cells were compared. In basal and activated status, both mRNA and protein abundance of CXCL10 is higher in *T*_RM_ cells (Figure S2D, Supporting Information), which confirmed that *T*_RM_ cell is a predominant source of CXCL10 in the pancreas of NOD mice. Collectively, these data implicated that FABP4 potentiates CXCL10 production from *T*_RM_ cells.

To further verify whether CXCL10 is the key mediator of FABP4‐regulated *T*_RM_ cell alarming function, FABP4^+/+^ *T*_RM_ and FABP4^−/−^ *T*_RM_ cells were subjected to recombinant CXCL10 protein stimulation or silencing of CXCL10 in the above‐mentioned co‐culture experiment for evaluating CD8^+^ T cell motility. Treatment with CXCL10 increased the motility of CD8^+^ T cells cultured in the conditioned medium of both FABP4^+/+^ *T*_RM_ and FABP4^−/−^ *T*_RM_ cells (Figure 4E), as indicated by the induction in track displacement length (2–3 folds) and average speed of CD8^+^ T cells (1.5–2 folds) (Figure 4F). Migration assay further demonstrated that CXCL10 increased the efficiency of CD8^+^ T cell migration when cultured in the conditioned medium of either FABP4^+/+^ *T*_RM_ and FABP4^−/−^ *T*_RM_ cells (Figure 4G; Figure S2E, Supporting Information). On the contrary, the expression and secretion of CXCL10 were suppressed in both FABP4^+/+^ *T*_RM_ and FABP4^−/−^ *T*_RM_ cells by siRNA knockdown approach (Figure S2F,G, Supporting Information). The motility of CD8^+^ T cells was significantly blunted when cells were cultured in the conditioned medium of siCXCL10‐treated FABP4^+/+^ *T*_RM_ and reached a level comparable to that of FABP4^−/−^ *T*_RM_ cells (Figure 4H). There was also a reduction in track displacement length (≈5 folds) and average speed (≈3 folds) of CD8^+^ T cells in the conditioned medium of CXCL10‐silenced FABP4^+/+^ *T*_RM_ cells compared to si‐Control treated cells. However, these parameters were not significantly altered in CD8^+^ T cells cultured in the conditioned medium of FABP4^−/−^ *T*_RM_ cells with or without CXCL10 silencing (Figure 4H,I). Consistently, the migration of CD8^+^ T cells substantially declined when cultured in the conditioned medium of siCXCL10‐treated *T*_RM_ cells (Figure 4J; Figure S2H, Supporting Information). Taken together, this evidence demonstrated that FABP4 aggravates the alarming function of *T*_RM_ cells via enhancing the expression of CXCL10, which promotes the recruitment of CD8^+^ T cells into the pancreas for auto‐antigen clearance, indicating that CXCL10 is a key mediator of the alarming function of *T*_RM_ cell.

### 2.5 Targeting FABP4 Resembles *T*_RM_ Cell Depletion in Alleviating T1D Development

**Figure 5:**
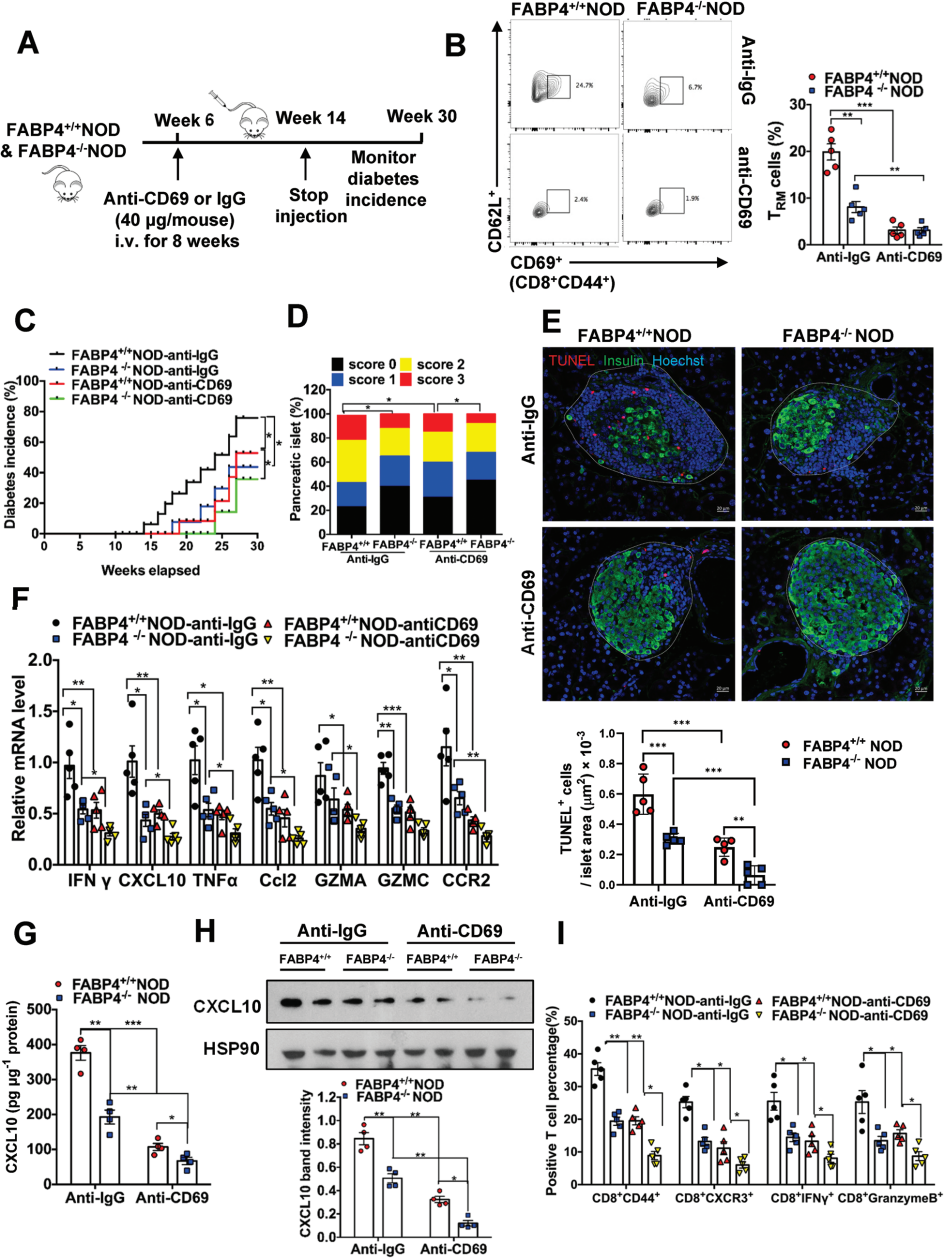
Targeting FABP4 resembles *T*_RM_ cell depletion in alleviating T1D development. FABP4^+/+^ NOD and FABP4^−/−^ NOD mice were subjected to the treatment of anti‐CD69 neutralizing antibody to deplete *T*_RM_ cells. A) Schematic diagram showing the protocol of *T*_RM_ cell depletion in NOD mice. Six‐week‐old female FABP4^+/+^ NOD and FABP4^−/−^ NOD mice were injected with anti‐CD69 antibody (40 µg/mouse) or IgG every week (i.v.) for 8 weeks (*n* = 5). B) The representative FACS plots show the abundance of *T*_RM_ cells (CD8^+^CD44^+^CD69^+^CD62L^−^) in the mouse pancreas. The right panel is the quantification of the percentage of *T*_RM_ cells (*n* = 5). C) Diabetes incidence of mice (*n* = 5). D) Scoring of histological grades of insulitis in mice (*n* = 5). E) Representative images of immunofluorescent co‐staining for insulin‐positive β cells (green) and TUNEL‐positive apoptotic cells (red) in mouse pancreas (scale bar 20 µm). The lower panel is the quantification of the number of apoptotic cells within the islet area (*n* = 5). F) The mRNA abundance of inflammatory cytokines and chemokines in mouse pancreas (*n* = 5). G) The abundance of CXCL10 protein in mouse pancreatic islets (*n* = 4). H) Representative immunoblots of CXCL10 and HSP90 in mouse pancreas. The lower panel is the quantification of the band intensity of CXCL10 relative to HSP90 (*n* = 4). I) The abundance of total cytotoxic T cells (CD8^+^CD44^+^), antigen‐experienced T cells (CD8^+^CXCR3^+^), and IFN γ or Granzyme B expressing CD8^+^ T cells infiltrated in the pancreatic islets of the above mice (*n* = 5). Data are expressed as mean ± SD. ^*^ *p* < 0.05, ^**^ *p* < 0.01, ^***^ *p* < 0.001. ANOVA followed by Sidak's test was used in (B), (E), (F), (G), (H), and (I). The log‐rank test was used in (C). The Pearson's chi‐square test was used in (D).

Taking account of the important function of FABP4 in *T*_RM_ cells, we next compared the suppressive effect of FABP4 deletion and *T*_RM_ cell depletion in alleviating T1D progression. As early myeloid cell activation occurred in islets of 2‐week‐old NOD mice, while adaptive immunity T cell activation initiated from 8 weeks (Figure S3A,B, Supporting Information), 6‐week‐old FABP4^+/+^ NOD and FABP4^−/−^ NOD mice were subjected to anti‐CD69 neutralizing antibody injection or IgG as control to subsequently deplete *T*_RM_ cells before T cell recruitment (**Figure** **5**A).

At the end of the monitoring period at week 30, *T*_RM_ cells were barely detected in the pancreas of both FABP4^+/+^ NOD and FABP4^−/−^ NOD mice receiving anti‐CD69 neutralizing antibody indicating the efficiency of *T*_RM_ cell depletion (Figure 5B). In *T*_RM_ cell‐depleted FABP4^+/+^ mice, diabetic onset was significantly postponed to 19 weeks old when compared to that of FABP4^+/+^ mice receiving IgG, which occurred from 14 weeks old. Notably, diabetic onset in FABP4^−/−^ mice receiving IgG control was observed from 18 weeks old which was similar to that of *T*_RM_ cell‐depleted FABP4^+/+^ mice (Figure 5C). Consistently, the diabetes incidence in both *T*_RM_ cell depleted FABP4^+/+^ NOD mice (52.85%) and FABP4^−/−^ NOD mice receiving IgG (43.73%) at 30 weeks old were significantly attenuated when compared to FABP4^+/+^ NOD mice receiving IgG (75.83%) (Figure 5C). The diabetic score of insulitis and pancreatic cell apoptosis as indicated by co‐staining of insulin and TUNEL was also reduced in both FABP4^−/−^ NOD mice with IgG and *T*_RM_ cell depleted FABP4^+/+^ mice (Figure 5D,E). These data suggest that FABP4 deletion and *T*_RM_ cell depletion delayed the T1D onset to a similar extent.

In addition to the inflammatory cytokines TNFα, Ccl2, GZMA, GZMC, and CCR2, the expression of IFNγ and CXCL10 was also significantly reduced (≈50%) in both FABP4^−/^ NOD mice receiving IgG and *T*_RM_ cell depleted FABP4^+/+^ mice (Figure 5F). A similar trend was observed in the protein abundance of CXCL10 in their pancreatic islets and whole tissue lysate (Figure 5G,H). Consistently, the population of total cytotoxic T cells (CD8^+^CD44^+^), the infiltration of antigen‐experienced T cells (CD8^+^CXCR3^+^), and the number of IFNγ expressing‐ and granzyme B expressing‐ CD8^+^ cells in the pancreas of both *T*_RM_ cell‐depleted FABP4^+/+^NOD and FABP4^−/−^NOD mice receiving IgG were significantly reduced, to a similar extent, comparing to that of FABP4^+/+^NOD mice receiving IgG (Figure 5I). All these findings supported that inhibition of FABP4 exhibited a similar protective effect as *T*_RM_ deletion in T1D progression. Notably, FABP4 deletion plus *T*_RM_ cell depletion exhibited a synergistic effect on suppression of T1D progression in mice as indicated by their diabetic incidence, pancreatic injury, expression of CXCL10, and T cell infiltration profile (Figure 5C–I), which highlights the pathogenic effect of both FABP4 and *T*_RM_ cells. Collectively, these data confirmed that targeting FABP4 is a potential therapeutic strategy for alleviating *T*_RM_ cell‐mediated adaptive immunity activation. Remarkably, the findings emphasize the importance of FABP4 as a key mediator facilitating the crosstalk between *T*_RM_ cells and cytotoxic T cells in T1D pathogenesis.

## 3 Conclusion 

**Figure 6:**
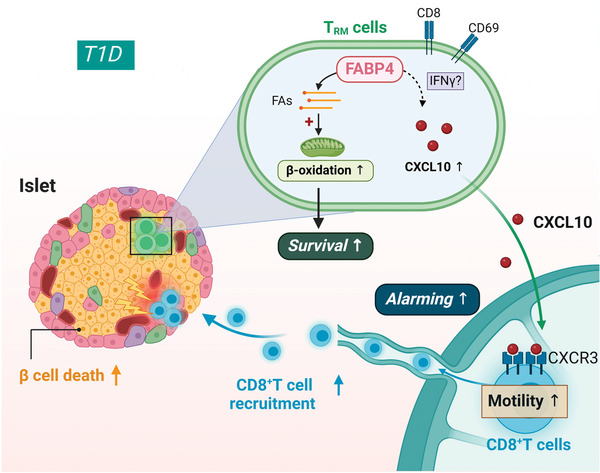
*T*_RM_ cells orchestrate the immunopathogenesis of type 1 Diabetes through FABP4‐CXCL10 axis.

Even though emerging evidence shows the substantial contribution of innate immunity to β cell destruction in the early phase of T1D,^[^ ^3^ ^]^ the pivotal role of adaptive immunity in T1D progression cannot be neglected. Considering the adverse effect of insulin treatment and the therapeutic potential of targeting immune cells as implicated in animal studies, identifying the crosstalk between immune cells in the pancreas is of critical importance for developing effective therapeutic strategies for this disease. The present study demonstrates, for the first time, that the *T*_RM_ cell is a novel player triggering the adaptive immune activation in T1D, which also expands the understanding of CD8^+^ T recruitment other than triggering by autoantigens released from β cells. Moreover, FABP4 was identified as a key mediator modulating both the survival and alarming function of *T*_RM_ cells in the pancreas, thus promoting the recruitment of cytotoxic CD8^+^ T cells into pancreatic islets and exaggerating insulitis and development of T1D in NOD mice (**Figure** **6**).

During the progression of T1D, CD8^+^CD69^+^ memory T cells accumulated in the distressed pancreatic islets and persisted as tissue‐resident memory T cells (*T*_RM_). Elevated FABP4 enhances *T*_RM_ survival by facilitating the uptake of fatty acids (FAs) and coordinating the fatty acid utilization in mitochondria. Meanwhile, FABP4 strengthens the alarming function of *T*_RM_ cells by upregulating CXCL10 production. Released CXCL10 in the microenvironment further activates CXCR3 on cytotoxic CD8^+^ T cells in lymph nodes (LNs) thus initiating the recruitment of cytotoxic T cells into pancreatic islets to potentiate insulitis and T1D progression.

## 4 Discussion

In the complex immunopathogenic mechanisms of T1D, there are three prerequisites: First, the β cell‐reactive T cells must become active. Second, the immune response must be pro‐inflammatory. Lastly, the immune system must fail to regulate these self‐attacking responses.^[^ ^27^ ^]^ Previous studies on T1D pathogenesis showed that β cells with epigenetic alternation or virus infection abnormally express MHC‐I and MHC‐II antigens initiating the activation of cytotoxic CD8^+^ T cells and dendritic cell‐mediated induction of anergic CD4^+^ T cells, respectively, whereby trigger the onset of adaptive immunopathogenesis.^[^ ^28^ ^]^ In the present study, we identified the CD8^+^CD69^+^ *T*_RM_ subset, which was account for a substantial proportion of the pancreatic T cells in patients with recent‐onset T1D,^[^ ^5^ ^]^ plays an essential role in the activation and perpetuation of adaptive immunity during T1D progression. We demonstrated that 1) CD8^+^CD69^+^ *T*_RM_ cells start accumulating in the injured islets of NOD mice of 6 weeks old; 2) *T*_RM_ cells promote the motility of cytotoxic CD8^+^ T cells and 3) elimination of *T*_RM_ cells delayed the onset of diabetic incidence from age 14 weeks to 19 weeks in NOD mice. Together with the attenuation in inflammatory cytokine production and cytotoxic CD8^+^ T cell recruitment in the pancreas, our findings reveal the fundamental role of *T*_RM_ cells in adaptive immunity during T1D progression and shed light on the therapeutic potential of manipulating *T*_RM_ cell activities.

The expression of FABP4 in different immune cell lineages exerts distinct pathogenic effects and has been implicated in various inflammatory diseases.^[^ ^29^ ^]^ In macrophages, FABP4 is a well‐known mediator of inflammatory response by enhancing inflammatory cytokines production.^[^ ^19^ ^]^ In dendritic cells (DCs), FABP4 mediates the secretion of selective cytokines such as IL‐12 and TNF thereby modulating the T cell priming.^[^ ^30^ ^]^ In T1D, we showed that in pancreatic islets, elevated FABP4 in macrophages enhances proinflammatory M1 subtype polarization thus creating an inflammatory milieu required for the activation of diabetogenic CD8^+^ T cells and shifting of CD4^+^ helper T cells toward Th1 subtypes, which implicate the critical role of FABP4 in facilitating the crosstalk between innate and adaptive immune cells.^[^ ^15^ ^]^ In this study, a significantly higher expression of FABP4 was observed in pancreatic CD8^+^ *T*_RM_ cells compared to other adaptive immune cell lineages and FABP4 promoted fatty acid utilization in pancreatic *T*_RM_ cells for prolonging their survival. These data are consistent with the findings showing the highest expression of FABP4 in skin *T*_RM_ cells among native T cells and various memory T cell types^[^ ^13^ ^]^ and FABP4 promotes survival of skin *T*_RM_ cells^[^ ^13^ ^]^ and cancer cells in gastric adenocarcinoma^[^ ^17^ ^]^ and colon cancer.^[^ ^31^ ^]^ Congruent with the high circulating fatty acid levels in T1D patients resulting from defective insulin‐mediated suppression of lipolysis,^[^ ^32^ ^]^ this finding reinforces the pathogenic significance of fatty acids in prolonging *T*_RM_ cell‐mediated pancreatic autoimmune attack on top of their toxicity on β cell apoptosis. Regarding FABP4‐mediated fatty acid utilization in *T*_RM_ cells, a metabolic flux analysis will help identify the key metabolic pathways that are reprogrammed by FABP4, which will expand the understanding of key determinants responsible for the survival of *T*_RM_ cells. Moreover, our data also indicated, for the first time, that FABP4 potentiates the *T*_RM_ cell alarming function by upregulating IFNγ and CXCL10 secretion, which further induces the recruitment of cytotoxic CD8^+^ T cells into the pancreas. Apart from FABP4‐mediated *T*_RM_ cell survival and alarming function, the present study also illustrated that *T*_RM_ cells may serve as effector cytotoxic T cells in the pancreatic islets. *T*_RM_ cells have been reported to gain effector‐like properties when encountering pathogens or infected cells.^[^ ^18^ ^]^ Our data showed that FABP4 enhances the expression of immune response genes such as IFNγ, CCL2, granzyme A, and granzyme C in *T*_RM_ cells. As IFNγ can induce β cell apoptosis^[^ ^20^ ^]^ and granzymes are key mediators of effector CD8^+^ T cell cytotoxicity,^[^ ^33^ ^]^ the finding indicating *T*_RM_ cells accumulated in pancreatic islets may potentially induce β cell apoptosis and FABP4 is the key determinant awakening this effect. However, the notion of *T*_RM_ cell‐mediated β cell death requires further investigations. Generally, therapeutic strategies through modulating FABP4 expression, fatty acids levels, or CXCL10 production may be effective for T1D with the elimination of *T*_RM_ cell‐mediated cytotoxic T cell recruitment and the subsequent β cell death.

Numerous factors derived from injured islets potentiate autoimmunity. Emerging clinical and animal studies emphasize the decisive role of islet‐derived CXCL10 in T1D progression and its therapeutic potential.^[^ ^34^, ^35^ ^]^ Previous studies have suggested that pancreatic lymph nodes and residual β cells are the potential sources of elevated CXCL10 in circulation and inflamed islets in recent onset T1D patients.^[^ ^26^, ^36^ ^]^ Another study showed a significant accumulation of *T*_RM_ cells in the insulitis pancreatic islets of recent onset T1D patients, which was associated with increased CXCL10 gene expression.^[^ ^5^ ^]^ In the present study, *T*_RM_ cells were identified as a novel source of CXCL10 in distressed islets of NOD mice. In a comprehensive comparison between human and mouse immune cells, the genetic patterns of most genes with similar functions are generally comparable across corresponding cell types in both species, with only a few hundred genes displaying divergent expression.^[^ ^37^ ^]^ In the development of T1D, the autoreactive response of CD4^+^ and CD8^+^ T cells to islet antigen in NOD mice closely recapitulates the pathogenesis of the disease in humans.^[^ ^38^ ^]^ Although human and mouse *T*_RM_ cells show discrepancies in tissue distribution, they share several similarities in terms of distinguishing features, genetic profiles, and their role in immunity.^[^ ^39^ ^]^ *T*_RM_ cells in both species can be identified using surface markers such as CD69 and CD103.^[^ ^39^ ^]^ Transcriptional profiling has shown that human spleen and lung *T*_RM_ cells, as well as mouse skin and gut *T*_RM_ cells, exhibit similarities in gene expression patterns.^[^ ^40^ ^]^ Moreover, both human and mouse *T*_RM_ cells play a role in virus control upon infection, highlighting their general importance in immunity.^[^ ^39^ ^]^ Considering the expression of FABP4 in human CD8^+^ *T*_RM_ cells^[^ ^13^ ^]^ and the expression of CXCL10 in mouse *T*_RM_ cells in the epidermis and dermis,^[^ ^41^ ^]^ it is highly possible that FABP4‐CXCL10 axis in pancreatic *T*_RM_ cells also plays a significant role in human T1D development. Further investigations are warranted to elucidate this potential relevance. CXCL10 has been reported to react with its receptor, CXCR3, on differentiated CD4^+^ T cells (Th1 cells) thus promoting the proinflammatory cytokine expression which favors the CD8^+^ T cells on destroying β‐cells.^[^ ^34^ ^]^ CXCL10 also activates CXCR3 on β cells in an autocrine manner and suppresses β cell proliferation.^[^ ^35^ ^]^ In the present study, we uncovered the important function of *T*_RM_ cell‐derived CXCL10 in enhancing CD8^+^ cytotoxic T cell motility, which promotes T cell infiltration in injured islets and aggravates β cell death. A comprehensive investigation using FABP4^−/−^ and FABP4^+/+^ NOD mice supplemented with CXCL10 neutralizing antibody or CXCL10 recombinant protein will allow a further understanding of the importance of FABP4‐CXCL10 axis in cytotoxic T cell recruitment in T1D progression. Besides, CXCL10 is known as IFNγ‐inducible protein 10 (IP‐10).^[^ ^42^ ^]^ FABP4 deficiency represses both IFNγ and CXCL10 production in *T*_RM_ cells while FABP4 potentiates palmitic acid‐induced CXCL10 expression in liver sinusoidal endothelial cells by promoting NF‐κB/p65 nuclear translocation.^[^ ^43^ ^]^ Since NF‐κB is a key modulator of IFNγ‐induced CXCL10 expression,^[^ ^44^, ^45^, ^46^ ^]^ it is possible that FABP4 enhances IFNγ thus upregulating CXCL10 expression in *T*_RM_ cells or FABP4 and IFNγ synergistically activate NF‐κB to induce CXCL10 production. Taken together, our findings expand the knowledge of CXCL10 in T1D by showing that *T*_RM_ cell is a novel source of CXCL10 which mediates *T*_RM_ cell alarming function.

While the effect of FABP4 inhibition in human T1D remains unclear, findings from the present study and other groups suggest that targeting FABP4 could potentially alleviate T1D and other inflammatory diseases. Elevated circulating FABP4 was observed in both adults^[^ ^15^ ^]^ and children^[^ ^47^ ^]^ with T1D, and was identified as a key regulator in ketoacidosis under T1D conditions.^[^ ^48^ ^]^ The pathogenic FABP4‐CXCL10 axis identified in the present study is aligned with clinical findings showing significant induction of CXCL10 in the circulation and inflamed islets of recent‐onset T1D patients.^[^ ^26^, ^36^ ^]^ CXCL10 not only enhances cytotoxic T cell recruitment but also promotes Th1 cell activation^[^ ^34^ ^]^ and suppresses β cell proliferation,^[^ ^35^ ^]^ thereby exacerbating T1D. FABP4 is a well‐known inflammatory regulator involved in various inflammatory diseases^[^ ^29^ ^]^ such as ischemic stroke,^[^ ^49^, ^50^ ^]^ non‐alcoholic steatohepatitis,^[^ ^51^, ^52^ ^]^ and atherosclerosis^[^ ^53^, ^54^ ^]^ by activating the JNK, NF‐κB, and JAK2/STAT2 signaling pathways in macrophages. Instead of modulating these dispersedly expressed multifunctional transcriptional factors, targeting FABP4 is relatively safe, as FABP4 shows restrictive expression patterns being predominantly expressed in cells such as adipocytes, endothelial cells, and certain immune cells.^[^ ^29^ ^]^ In addition to the beneficial effect of pharmacological inhibitor of FABP4, BMS309403, in cardiometabolic diseases,^[^ ^29^ ^]^ the application of FABP4 neutralizing antibodies exhibited therapeutic potential in obesity‐related impairments in glucose metabolism and systemic inflammation.^[^ ^55^, ^56^, ^57^ ^]^ The findings about our newly developed anti‐FABP4 neutralizing monoclonal antibody 6H2 has demonstrated that targeting FABP4 is a viable therapeutic strategy to neutralize JNK/c‐Jun activation in macrophages and treat ischemic stroke.^[^ ^58^ ^]^ In 2022, a CD3‐directed antibody teplizumab was approved by the Food and Drug Administration (FDA) to delay the onset of stage 3 in T1D patients.^[^ ^59^ ^]^ This initial success not only implicates the potential of using antibody therapy in treating this disease but also highlights the importance of early intervention. Given that the present study identified FABP4 as an inflammatory regulator potentiating the *T*_RM_ cell‐mediated adaptive immune activation before T1D onset, the proven similarity of immunology and pathology of human T1D and NOD mouse model,^[^ ^38^ ^]^ and the metabolic benefits of FABP4 deletion,^[^ ^60^ ^]^ the application of FABP4 neutralizing antibody as early intervention is a potential low‐risk immunotherapy option to delay T1D progression. Moreover, considering the effect of FABP4 in prolonging *T*_RM_ cell survival, the pathogenic effect of *T*_RM_ cells identified in human autoimmune skin diseases,^[^ ^61^ ^]^ and the physiological role of *T*_RM_ cells in maintaining immune homeostasis,^[^ ^6^ ^]^ targeting FABP4 may help to modulate *T*_RM_ cell activity and offer a safety advantage over eliminating *T*_RM_ cells in treating these diseases. Further studies are needed to explore the translational potential of targeting FABP4.

Although the present study provides substantial evidence supporting the notion that FABP4 potentiates *T*_RM_ cell‐mediated autoimmune pathogenesis in T1D, some limitations must be noted. First, *T*_RM_ cell was identified as the major pancreatic source of CXCL10 while the contribution of other cell types such as monocytes,^[^ ^62^ ^]^ macrophages,^[^ ^63^ ^]^ and endothelial cells^[^ ^64^ ^]^ that express both CXCL10 and FABP4 cannot be excluded. The interregulation of FABP4 on CXCL10 production in these cells also warrants further investigations. Second, as a subset of circulating Treg cells also express CD69,^[^ ^65^ ^]^ depletion of *T*_RM_ cells using anti‐CD69 antibody in the present study may potentially eliminate some Treg cells. Nevertheless, the defective immunosuppressive effect in Treg cells was evidenced in both T1D patients and NOD mouse models.^[^ ^66^, ^67^ ^]^ In this case, although the usage of anti‐CD69 antibody to deplete *T*_RM_ cells was accompanied by the potential elimination of CD69^+^ Treg cells, the beneficial effect of CD69 neutralization in T1D is mainly attributed to *T*_RM_ cell depletion.

In summary, our present study uncovers a novel pathological role of *T*_RM_ cells in altering adaptive immune responses in pancreatic islets during the progression of T1D. *T*_RM_ cell depletion in NOD mice at a relatively late age (6 weeks old) was sufficient to ameliorate insulitis and attenuate the development of T1D. Taken together, our previous study showed that FABP4 mediates the innate immunity in macrophages of NOD mice as early as they are 2 weeks old and the present study identified that FABP4 regulates the survival and alarming function of *T*_RM_ cells. Thus, our findings suggest that targeting FABP4 is a potential therapeutic strategy for early or late management of autoimmune diabetes.

## 5 Experimental Section

### Animals

NOD/ShiLtJ mice were bought from Jackson's lab. FABP4 deficient (FABP4^−/−^) mice in C57BL/6N background were generated using the same procedures as previously described.^[^ ^68^ ^]^ FABP4^−/−^ mice were backcrossed with NOD/ShiLtJ mice for at least ten generations to obtain FABP4^+/+^ NOD mice and FABP4^−/−^ NOD mice. Age‐matched female FABP4^+/+^ NOD mice and FABP4^−/−^ NOD mice were used in all the experiments of this study. Animals were allocated to their experimental groups according to their genotypes. The investigators were not blinded to the experimental group. Mice were housed in a temperature‐controlled facility (23 °C, 12‐h light / dark cycle, 60–70% humidity). For depletion of *T*_RM_ cells, 6‐week‐old female FABP4^+/+^ NOD and FABP4^−/−^ NOD mice were subjected to the injection of anti‐CD69 neutralizing antibody (40 µg permouse, i.p., Abcam) or IgG control for 8 weeks and sacrificed at 30 weeks old (*n* = 5). All experimental protocols were approved by the Committee on the Use of Live Animals in Teaching and Research at the University of Hong Kong (4784‐18).

### Diabetes Diagnosis

The random blood glucose level of female FABP4^+/+^ NOD mice and FABP4^−/−^ NOD mice with anti‐CD69 neutralizing antibody or IgG treatment was monitored weekly using a glucose test meter (Roche ACCU‐CHEK) and strips (Roche ACCU‐CHEK) according to manufactory instruction. Overt diabetes was defined as two consensus‐positive blood glucose tests above 13.9 mmol L^−1^ (250 mg dL^−1^).^[^ ^69^ ^]^

### Isolation and Dissociation of Pancreatic Islets

Pancreas from age‐matched female FABP4^+/+^ NOD mice and FABP4^−/−^ NOD mice was perfused with collagenase P solution (1 mg mL^−1^, Roche), and subjected to digestion for 10 mins at 37 °C. The digestion was stopped by adding HBSS + 5% FCS followed by extensive washes with HBSS. The homogenates were then filtered through 500 and 70 µm cell strainers (BD Biosciences) subsequently. While the flow‐through fraction of the 500 µm strainer contains most exocrine cells, the fraction captured upon the 70 µm cell strainer is mostly composed of islets. The captured islets were further dissociated by a non‐enzymatic dissociation solution (Sigma–Aldrich) for 10 mins at room temperature.

### Flow Cytometry Analysis

The cell suspensions from dissociated pancreatic islets of age‐matched female NOD mice (*n* = 5) were subjected to antibody staining and flow cytometry analysis by BD LSR Fortessa Cell Analyzer (BD Biosciences). Briefly, isolated cells were subjected to red blood cell lysis and Live/dead fixable cell staining. For surface marker staining, cells were stained with the following antibodies: rat anti‐mouse CD3 (clone 17A2, Biolegend), rat anti‐mouse CD8a (clone 53–6.7, BD Biosciences), rat anti‐mouse CD44 (clone IM7, BD Biosciences), rat anti‐mouse CD62L (clone MEL‐14, Biolegend), Armenian hamster anti‐mouse CD69 (clone H1.2F3, Biolegend), Armenian hamster anti‐mouse CD103 (clone 2E7, Biolegend), or Armenian hamster anti‐mouse CXCR3 (clone CXCR3‐173, Biolegend). For analysis of Ki67, *T*_RM_ cells were fixed and permeabilized using the Fixation/Permeabilization kit (eBioscience) according to the manufacturer's instructions before antibody staining with rat anti‐mouse Ki67 (clone 16A8, Biolegend). For intracellular cytokine staining, cells were cultured in RPMI 1640 medium supplemented with 10% FBS and 1% PSF and stimulated with phorbol 12‐myristate 13‐acetate (PMA, 50 ng mL^−1^, Sigma‐Aldrich) and ionomycin (1 µg mL^−1^, Sigma‐Aldrich) in the presence of GolgiStopTM protein transport inhibitor (BD Biosciences). After stimulation for 5 hrs, cells were collected and surface stained with rat anti‐mouse CD8a (clone 53–6.7, Biolegend), Armenian hamster anti‐mouse CD69 (clone H1.2F3, Biolegend), and fixed in 2% PFA for 25 mins on ice. Cells were then permeabilized with 0.2% saponin/PBS and subjected to the staining with the following antibodies: rat anti‐mouse IFNγ (clone XMG1.2, BD Biosciences), mouse anti‐human/mouse granzyme B (clone QA16A02, Biolegend), rabbit anti‐mouse CXCL10 (clone 10H11L3, Invitrogen) and its relative secondary antibody goat anti‐rabbit‐PE (Santa Cruz). Data were analyzed using Flow Jo software version X.0.7 (Tree Star, Inc.).

### Fractionation of Immune Cells from Pancreatic Islet

B cells and various types of T cells (*T*_N_, *T*_eff_, *T*_CM_, *T*_EM_, *T*_RM_) were isolated from dissociated pancreatic islets of 10‐week‐old female FABP4^+/+^ NOD mice and FABP4^−/−^ NOD mice (*n* = 5) by fluorescence‐activated cell sorting (FACs). Isolated cells with purity above 95% were further subjected to experiments. Briefly, the cells from dissociated islets were first incubated in red blood cell lysis buffer (BioLegend) for 5 min on ice and re‐suspended in 1 × PBS buffer. Subsequently, cells were subjected to live/dead fixable cell staining (Invitrogen) for 30 mins on ice. Afterward, cells were washed once with FACS buffer (1% BSA in 1 × PBS) followed by staining with the following antibodies: rat anti‐mouse CD19 (clone 1D3, BD Biosciences), rat anti‐mouse CD3 (clone 17A2, BD Biosciences), rat anti‐mouse CD8a (clone 53–6.7, BD Biosciences), rat anti‐mouse CCR7 (clone 4B12, Biolegend) rat anti‐mouse CD44 (clone IM7, BD Biosciences), rat anti‐mouse CD62L (clone MEL‐14, Biolegend), and Armenian hamster anti‐mouse CD69 (clone H1.2F3, Biolegend). FACs were performed by using BD FACSAria SORP (BD bioscience). Voltages were adjusted based on unstained cells and compensation was set using single‐stained positive controls for each color. The cell types were defined with their specific surface markers: B cell (CD3^−^CD19^+^), T_N_ (CD3^+^CD8^+^CCR7^+^), *T*_eff_ (CD3^+^CD8^+^CCR7^−^), *T*_CM_ (CD8^+^CD44^+^CD69^−^CD62L^+^), *T*_EM_ (CD8^+^CD44^+^CD69^−^CD62L^−^), *T*_RM_ (CD8^+^CD44^+^CD69^+^CD62L^−^), respectively.

### *T*_RM_ Cell Survival Analysis and Fatty Acid Uptake

FABP4^+/+^ *T*_RM_ and FABP4^−/−^ *T*_RM_ cells (CD8^+^CD44^+^CD69^+^CD62L^−^) were sorted from the pancreatic islet of NOD mice and labeled with carboxyfluorescein succinimidyl ester (CFSE, 65–0850; eBioscience) then transferred intravenously into female recipient mice with a total number of 3 × 10^6^ cells. Mice were anesthetized and imaged using PE IVIS Spectrum in vivo imaging system (PerkinElmer) after adoptive transfer on day 1, day 5, and day 20 (*n* = 5). For the in vitro experiment, to evaluate the spontaneous apoptosis of FABP4^+/+^ *T*_RM_ and FABP4^−/−^ *T*_RM_ cells, cells were cultured in a T cell culture medium (AIM V Medium, Gibco) for 72 hrs, cells were harvested and stained with 7‐AAD (BD science) for flow cytometry analysis. For fatty acid uptake in *T*_RM_ cells, BODIPY‐FA (5 μM, Molecular Probes) was added to *T*_RM_ cells for 10 mins. The flow cytometry analysis was conducted using BD LSR Fortessa Cell Analyzer (BD Biosciences).

### *T*_RM_ Cell Expansion and Activation

FABP4^+/+^ *T*_RM_ and FABP4^−/−^ *T*_RM_ cells (CD8^+^CD44^+^CD69^+^CD62L^−^) were sorted from the pancreas of NOD mice and cultured in a special T cell culture medium (AIM V Medium, Gibco) with the presence of recombinant IL‐2 (30U mL^−1^, 130‐097‐742, Miltenyi Biotec). Cells were placed in a 24‐well plate (2 mL per well) in an incubator with 37 °C, 5% CO_2_, and 95% humidity. Cells were split every two to three days, each plate should have at least ten million T cells after 6–7 days by the end of the expansion. Stimulation was achieved by the addition of Dynabeads Mouse T‐Activator CD3/CD28 (5 mg mL^−1^, 11456D, Gibco) for 3 days. The conditioned medium was collected to analyze the concentration of IFNγ and CXCL10 to evaluate the immune response of FABP4^+/+^ *T*_RM_ and FABP4^−/−^ *T*_RM_ cells or the recall ability of CD8^+^ T cells in the migration experiment.

### Cell Migration

CD8^+^ T cells were isolated from the spleen of NOD mice and purified using CD8a (Ly‐2) MicroBeads (130‐117‐044, Miltenyi Biotec) according to the manufacturer's protocols. Cells were then expanded and activated by Dynabeads Mouse T‐Activator CD3/CD28 (11456D, Gibco) in the presence of 30 U mL^−1^ of recombinant human IL‐2 in the TexMACS medium (Miltenyi Biotec) until confluence.^[^ ^25^ ^]^ Trans‐wells with 5 mm pore size (Costar) were coated overnight with RetroNectin (Takara Bio) at 50 mg mL^−1^. Activated CD8^+^ T cells (1 × 10^5^ cells/well) were then seeded on the porous permeable membrane in the Trans‐well Supports (Corning). To assess the alarming function of FABP4^+/+^ *T*_RM_ and FABP4^−/−^ *T*_RM_ cells and the involvement of CXCL10, the conditioned medium of respective cells were added to the lower wells. and supplemented with recombinant murine CXCL10 (2 g mL^−1^, PeproTech) or IgG as control. T cells were allowed to migrate through the Trans‐well membrane for 3 hrs at 37 °C. Migrated cells were collected and labeled with counting beads (AccuCheck Counting Beads Kit, Invitrogen). The cell number was assessed by flow cytometry counting with BD LSR Fortessa Cell Analyzer (BD Biosciences). Data were analyzed using FlowJo software version X.0.7 (Tree Star, Inc.).

### CXCL10 Silencing

CXCL10 knockdown *T*_RM_ cell was obtained by transfecting *T*_RM_ cells with CXCL10 siRNA (siCXCL10, Integrated DNA Technologies ) for 24 hrs. Cells transfected with scramble siRNA (siCtrl) (Integrated DNA Technologies ) served as control cells. The transfection efficiency was evaluated by the protein expression of CXCL10 in *T*_RM_ cells and the concentration of CXCL10 in the conditioned medium.

### Motility of Cytotoxic T Cells

Freshly isolated CD8^+^ T cells were expanded and activated as above mentioned to obtain cytotoxic T cells. Cells were then infected with GFP lentivirus (1 × 10^5^ IFU mL^−1^). Subsequently, CD8^+^ T cells were seeded on ICAM‐1–coated chambered coverslips and incubated at 37 °C for 1 hr to let the cells recover and adhere. Afterward, the cells were washed three times to remove the non‐adhered cells. Then, the chambered coverslips were mounted on an inverted microscope connected to a Cell Observer (Zeiss) and analyzed with a 340 objective and oil immersion, as well as with full temperature and CO_2_ incubation during image acquisition. Conditioned medium of FABP4^+/+^ *T*_RM_ and FABP4^−/−^ *T*_RM_ cells supplemented with recombinant CXCL10 or IgG or FABP4^+/+^ *T*_RM_ and FABP4^−/−^ *T*_RM_ cells treated with siCXCL10 or siCtrl were directly added to the cell mixture during acquisition. The images were taken at ten frames per second using a fluorescence microscope (QImaging, Olympus IX71).

### Measurement of Cellular OCR

Cellular OCR was measured using the XFe24 Extracellular Flux Analyser (Seahorse Bioscience). FABP4^+/+^ *T*_RM_ and FABP4^−/−^ *T*_RM_ cells were seeded in an XFe24‐well microplate. Cells were treated with palmitic acid (PA, 200 nM) with or without pre‐incubation with BSA (0.3%) for 30 mins. The OCR was measured under basal conditions and in response to 1 µMOligomycin (ATP synthase inhibitor, Sigma–Aldrich), 1.5 µM carbonyl cyanide‐4‐ (trifluoromethoxy) phenylhydrazone (FCCP, cellular uncoupler, Sigma–Aldrich), 100 nM rotenone & 1 µM antimycin A (Sigma–Aldrich) to sequentially determine the basal‐, ATP‐dependent‐, maximal‐ and mitochondria‐independent oxygen consumption, respectively.^[^ ^13^ ^]^

### Immunofluorescence (IF) Staining

The frozen pancreas sections were prepared at a thickness of 8 µm. Acetone‐fixed sections were incubated with primary antibody against insulin (A2090, ABclonal), FABP4 (AF1443, R&D Systems), or CD69 (#104 502, clone H1.2F3, Biolegend). Subsequently, Alexa Fluor 488, 594, or 647‐labeled secondary antibodies were applied accordingly. DNA was counterstained with Hoechst 33342 (#62249, Thermo Scientific). The paraffin pancreas sections were prepared at a thickness of 5 µm. Deparaffinized sections were subjected to TUNEL staining (A112‐01, Vazyme) according to the manufacturer's instructions. Afterward, the sections were subjected to the staining of insulin as above mentioned. The sections were visualized under the ZEISS LSM800 Confocal Microscope.

### Immunoblot Analysis

Proteins were separated by SDS–polyacrylamide gel electrophoresis, transferred to polyvinylidene difluoride membranes and probed with primary antibodies against CXCL10 (0.5 mg mL^−1^, rabbit monoclonal; 701225, Invitrogen), and HSP90 (0.25 mg mL^−1^, rabbit monoclonal; 4877, Cell Signaling). The intensities of protein bands were quantified using the NIH Image J software.

### Quantitative Real‐Time PCR

Total RNA was extracted with RNAiso Plus (Takara) according to the manufacturer's instructions. Complementary DNA was prepared using PrimeScript RT Reagent Kit (Takara). Quantitative real‐time PCR was performed using SYBR Premix Ex Taq (Takara) on a 7900 HT PCR Machine (Applied Biosystems). The relative mRNA expression of a specific gene was measured with 2‐ΔΔCt method and normalized against the house‐keeping gene β‐actin. All the primers were purchased from Invitrogen.

### Immunological Analysis

The concentration of mouse CXCL10 and IFNγ in the cell‐conditioned medium was determined with commercial enzyme‐linked immunosorbent assay (ELISA) kits (BMS6018 and # 88‐7314‐88, Invitrogen) according to the manufacturer's instructions. For ELISA measurements of CXCL10 or IFNγ in pancreatic islets or mouse pancreas, fresh‐frozen samples were homogenized in buffer containing 1M  NaCl, 10 mM HEPES (pH 7.4), and 0.5% Triton X‐100 with the cocktail of protease inhibitors (Roche). The protein concentration of CXCL10 and IFNγ in the protein lysates was quantified with the immunoassay as mentioned above and normalized against protein abundance of lysate of pancreatic islets or tissue weight.

### Statistical Analysis

All the replicate experiments including in vivo and in vitro experiments were repeated at least two times. All statistical analyses were performed using GraphPad Prism 8.0 software (San Diego, CA, USA). All data are expressed as mean ± SD. No data were excluded from the statistical analysis. The animal sample size for each study was chosen based on literature documentation of similar well‐characterized experiments.^[^ ^15^ ^]^ The diabetes incidence was diagrammed according to the Kaplan–Meier method and incidences between different groups were compared with the log‐rank test. Data normality was accessed by the Shapiro‐Wilk test. Differences between the two groups were evaluated using the Student's *t* test for normal distributions or the Mann‐Whitney *U* test for non‐normal distributions. Differences between multiple groups were compared using ANOVA followed by Sidak's test. The difference in insulitis scores among groups was compared using Pearson's chi‐square test. All statistical tests were two‐tailed. *p*‐values <0.05 were considered to indicate statistically significant differences. Representation of the *p*‐value was ^*^ *p* < 0.05, ^**^ *p* < 0.01, ^***^ *p* < 0.001.

## Conflict of Interest

The authors declare no conflict of interest.

## Author Contributions

R.L.C.H., and L.S. contributed equally to this work. L.S. designed the study; X.W., L.S., L.C., L.Y., L.J., Z.Z., and Y.X. conducted the experiments and analyzed the data; L.S. and X.W. prepared the figures and drafted the manuscript; L.S., Z.G.Z., A.X., and R.L.C.H. edited the manuscript.

## Supporting information

Supporting Information

## Data Availability

The data that support the findings of this study are available from the corresponding author upon reasonable request.

## References

[1] M. A. Atkinson , G. S. Eisenbarth , A. W. Michels , Lancet. 2014, 383, 69. 23890997 10.1016/S0140-6736(13)60591-7 PMC4380133

[2] L. A. DiMeglio , C. Evans‐Molina , R. A. Oram , Lancet. 2018, 391, 2449. 29916386 10.1016/S0140-6736(18)31320-5 PMC6661119

[3] A. Lehuen , J. Diana , P. Zaccone , A. Cooke , Nat. Rev. Immunol. 2010, 10, 501. 20577267 10.1038/nri2787

[4] L. Sun , S. Xi , G. He , Z. Li , X. Gang , C. Sun , W. Guo , G. Wang , J. Diabetes Res. 2020, 2020, 4106518. 32802890 10.1155/2020/4106518 PMC7415089

[5] E. Kuric , P. Seiron , L. Krogvold , B. Edwin , T. Buanes , K. F. Hanssen , O. Skog , K. Dahl‐Jørgensen , O. Korsgren , Am. J. Pathol. 2017, 187, 581. 28212742 10.1016/j.ajpath.2016.11.002

[6] S. P. Weisberg , D. J. Carpenter , M. Chait , P. Dogra , R. D. Gartrell‐Corrado , A. X. Chen , S. Campbell , W. Liu , P. Saraf , M. E. Snyder , M. Kubota , N. M. Danzl , B. A. Schrope , R. Rabadan , Y. Saenger , X. Chen , D. L. Farber , Cell Rep. 2019, 29, 3916. 31851923 10.1016/j.celrep.2019.11.056 PMC6939378

[7] L. K. Mackay , A. Braun , B. L. Macleod , N. Collins , C. Tebartz , S. Bedoui , F. R. Carbone , T. Gebhardt , J. Immunol. 2015, 194, 2059. 25624457 10.4049/jimmunol.1402256

[8] A. Zaid , J. L. Hor , S. N. Christo , J. R. Groom , W. R. Heath , L. K. Mackay , S. N. Mueller , J. Immunol. 2017, 199, 2451. 28855310 10.4049/jimmunol.1700571

[9] R. A. Clark , Sci. Transl. Med. 2015, 7, 269rv1. 10.1126/scitranslmed.3010641 PMC4425129 25568072

[10] S. N. Mueller , L. K. Mackay , Nat. Rev. Immunol. 2016, 16, 79. 26688350 10.1038/nri.2015.3

[11] G. M. Tovar , C. Gallen , T. Bergsbaken , J. Immunol. 2024, 212, 361. 38227907 10.4049/jimmunol.2300399 PMC10794029

[12] M. F. Krummel , F. Bartumeus , A. Gerard , Nat. Rev. Immunol. 2016, 16, 193. 26852928 10.1038/nri.2015.16 PMC4869523

[13] Y. Pan , T. Tian , C. O. Park , S. Y. Lofftus , S. Mei , X. Liu , C. Luo , J. T. O'Malley , A. Gehad , J. E. Teague , S. J. Divito , R. Fuhlbrigge , P. Puigserver , J. G. Krueger , G. S. Hotamisligil , R. A. Clark , T. S. Kupper , Nature. 2017, 543, 252. 28219080 10.1038/nature21379 PMC5509051

[14] A. C. Wotherspoon , I. S. Young , D. R. McCance , C. C. Patterson , M. J. A. Maresh , D. W. M. Pearson , J. D. Walker , V. A. Holmes , Diabetes Care. 2016, 39, 1827. 27630211 10.2337/dc16-0803

[15] Y. Xiao , L. Shu , X. Wu , Y. Liu , L. Y. Cheong , B. Liao , X. Xiao , R. L. C. Hoo , Z. Zhou , A. Xu , JCI Insight. 2021, 6, e141814. 33690220 10.1172/jci.insight.141814 PMC8119222

[16] S. DeWolf , M. Sykes , J. Clin. Invest. 2017, 127, 2473. 28628037 10.1172/JCI90595 PMC5490749

[17] R. Lin , H. Zhang , Y. Yuan , Q. He , J. Zhou , S. Li , Y. Sun , D. Y. Li , H.‐B. Qiu , W. Wang , Z. Zhuang , B. Chen , Y. Huang , C. Liu , Y. Wang , S. Cai , Z. Ke , W. He , Cancer Immunol. Res. 2020, 8, 479. 32075801 10.1158/2326-6066.CIR-19-0702

[18] J. M. Schenkel , D. Masopust , Immunity. 2014, 41, 886. 25526304 10.1016/j.immuni.2014.12.007 PMC4276131

[19] X. Hui , H. Li , Z. Zhou , K. S. L. Lam , Y. Xiao , D. Wu , K. Ding , Y. Wang , P. M. Vanhoutte , A. Xu , J. Biol. Chem. 2010, 285, 10273. 20145251 10.1074/jbc.M109.097907 PMC2856232

[20] J. Barthson , C. M. Germano , F. Moore , A. Maida , D. J. Drucker , P. Marchetti , C. Gysemans , C. Mathieu , G. Nuñez , A. Jurisicova , D. L. Eizirik , E. N. Gurzov , J. Biol. Chem. 2011, 286, 39632. 21937453 10.1074/jbc.M111.253591 PMC3234786

[21] J. N. Pardo , A. Bosque , R. Brehm , R. Wallich , J. Naval , A. Mu?llbacher , A. Anel , M. M. Simon , J. Cell Biol. 2004, 167, 457. 15534000 10.1083/jcb.200406115 PMC2172484

[22] H. Johnson , L. Scorrano , S. J. Korsemeyer , T. J. Ley , Blood. 2003, 101, 3093. 12515723 10.1182/blood-2002-08-2485

[23] A. Marshall , A. Celentano , N. Cirillo , M. McCullough , S. Porter , PLoS One. 2017, 12, e0172821. 28253295 10.1371/journal.pone.0172821 PMC5333845

[24] S. Tanaka , Y. Nishida , K. Aida , T. Maruyama , A. Shimada , M. Suzuki , H. Shimura , S. Takizawa , M. Takahashi , D. Akiyama , S. Arai‐Yamashita , F. Furuya , A. Kawaguchi , M. Kaneshige , R. Katoh , T. Endo , T. Kobayashi , Diabetes. 2009, 58, 2285. 19641142 10.2337/db09-0091 PMC2750208

[25] A. Trickett , Y. L. Kwan , J. Immunol. Methods. 2003, 275, 251. 12667688 10.1016/s0022-1759(03)00010-3

[26] T. Shigihara , Y. Oikawa , Y. Kanazawa , Y. Okubo , S. Narumi , T. Saruta , A. Shimada , J. Autoimmun. 2006, 26, 66. 16309891 10.1016/j.jaut.2005.09.027

[27] M. Wallberg , A. Cooke , Trends Immunol. 2013, 34, 583. 24054837 10.1016/j.it.2013.08.005

[28] J. Boldison , F. S. Wong , Trends Endocrinol. Metab. 2016, 27, 856. 27659143 10.1016/j.tem.2016.08.007

[29] H.‐L. Li , X. Wu , A. Xu , R. L. Hoo , Int. J. Mol. Sci. 2021, 22, 9386. 34502295 10.3390/ijms22179386 PMC8456319

[30] M. S. Rolph , T. R. Young , B. O. V. Shum , C. Z. Gorgun , C. Schmitz‐Peiffer , I. A. Ramshaw , G. S. Hotamisligil , C. R. Mackay , J. Immunol. 2006, 177, 7794. 17114450 10.4049/jimmunol.177.11.7794

[31] W. Tian , W. Zhang , Y. Zhang , T. Zhu , Y. Hua , H. Li , Q. Zhang , M. Xia , Cancer Cell Int. 2020, 20, 512. 33088219 10.1186/s12935-020-01582-4 PMC7574203

[32] I. E. Schauer , J. K. Snell‐Bergeon , B. C. Bergman , D. M. Maahs , A. Kretowski , R. H. Eckel , M. Rewers , Diabetes. 2011, 60, 306. 20978091 10.2337/db10-0328 PMC3012187

[33] J. A. Trapani , Genome Biol. 2001, 2, reviews30141. 10.1186/gb-2001-2-12-reviews3014 PMC138995 11790262

[34] A. Antonelli , S. M. Ferrari , A. Corrado , E. Ferrannini , P. Fallahi , Cytokine Growth Factor Rev. 2014, 25, 57. 24529741 10.1016/j.cytogfr.2014.01.006

[35] A. Shimada , Y. Oikawa , Y. Yamada , Y. Okubo , S. Narumi , Rev. Diabet. Stud. 2009, 6, 81. 19806237 10.1900/RDS.2009.6.81 PMC2779012

[36] S. Uno , A. Imagawa , K. Saisho , K. Okita , H. Iwahashi , T. Hanafusa , I. Shimomura , Endocr. J. 2010, 57, 991. 20966598 10.1507/endocrj.k10e-076

[37] T. Shay , V. Jojic , O. Zuk , K. Rothamel , D. Puyraimond‐Zemmour , T. Feng , E. Wakamatsu , C. Benoist , D. Koller , A. Regev , Proc. Natl. Acad. Sci. USA 2013, 110, 2946. 23382184 10.1073/pnas.1222738110 PMC3581886

[38] T. C. Thayer , S. B. Wilson , C. E. Mathews , Endocrinol. Metab. Clin. North Am. 2010, 39, 541. 20723819 10.1016/j.ecl.2010.05.001 PMC2925291

[39] P. A. Szabo , M. Miron , D. L. Farber , Sci. Immunol. 2019, 4, aas9673. 10.1126/sciimmunol.aas9673 PMC6778482 30952804

[40] B. V. Kumar , W. Ma , M. Miron , T. Granot , R. S. Guyer , D. J. Carpenter , T. Senda , X. Sun , S.‐H. Ho , H. Lerner , A. L. Friedman , Y. Shen , D. L. Farber , Cell Rep. 2017, 20, 2921. 28930685 10.1016/j.celrep.2017.08.078 PMC5646692

[41] J. M. Richmond , J. P. Strassner , M. Rashighi , P. Agarwal , M. Garg , K. I. Essien , L. S. Pell , J. E. Harris , J. Invest. Dermatol. 2019, 139, 769. 30423329 10.1016/j.jid.2018.10.032 PMC6431571

[42] A. Antonelli , S. M. Ferrari , D. Giuggioli , E. Ferrannini , C. Ferri , P. Fallahi , Autoimmun. Rev. 2014, 13, 272. 24189283 10.1016/j.autrev.2013.10.010

[43] C. Zhou , Z. Shen , B. Shen , W. Dai , Z. Sun , Y. Guo , X. Xu , J. Wang , J. Lu , Q. Zhang , X. Luo , Y. Qu , H. Dong , L. Lu , Biochim. Biophys. Acta Mol. Basis Dis. 2023, 1869, 166810. 37487374 10.1016/j.bbadis.2023.166810

[44] N. Tamassia , F. Calzetti , T. Ear , A. Cloutier , S. Gasperini , F. Bazzoni , P. P. McDonald , M. A. Cassatella , Eur. J. Immunol. 2007, 37, 2627. 17668902 10.1002/eji.200737340

[45] E. L. Hardaker , A. M. Bacon , K. Carlson , A. K. Roshak , J. J. Foley , D. B. Schmidt , P. T. Buckley , M. Comegys , R. A. Panettieri , H. M. Sarau , K. E. Belmonte , FASEB J. 2004, 18, 191. 14597565 10.1096/fj.03-0170fje

[46] S. Yeruva , G. Ramadori , D. Raddatz , Int. J. Colorectal Dis. 2008, 23, 305. 18046562 10.1007/s00384-007-0396-6 PMC2225996

[47] H. Liu , M. Guo , F. L. Jiang , J. Diabetes Complications. 2018, 32, 488. 29526626 10.1016/j.jdiacomp.2017.12.009

[48] N. Gruber , M. Rathaus , I. Ron , R. Livne , S. Sheinvald , E. Barhod , R. Hemi , A. Tirosh , O. Pinhas‐Hamiel , A. Tirosh , Diabetologia. 2022, 65, 366. 34806114 10.1007/s00125-021-05606-0

[49] B. Liao , L. Geng , F. Zhang , L. Shu , L. Wei , P. K. K. Yeung , K. S. L. Lam , S. K. Chung , J. Chang , P. M. Vanhoutte , A. Xu , K. Wang , R. L. C. Hoo , Eur. Heart J. 2020, 41, 3169. 32350521 10.1093/eurheartj/ehaa207 PMC7556749

[50] W. J. Tu , X. W. Zeng , A. Deng , S. J. Zhao , D. Z. Luo , G. Z. Ma , H. Wang , Q. Liu , Stroke. 2017, 48, 1531. 28487339 10.1161/STROKEAHA.117.017128

[51] R. L. C. Hoo , I. P. C. Lee , M. Zhou , J. Y. L. Wong , X. Hui , A. Xu , K. S. L. Lam , J. Hepatol. 2013, 58, 358. 23108115 10.1016/j.jhep.2012.10.022

[52] K.‐L. Milner , D. van der Poorten , A. Xu , E. Bugianesi , J. G. Kench , K. S. L. Lam , D. J. Chisholm , J. George , Hepatology. 2009, 49, 1926. 19475694 10.1002/hep.22896

[53] Y.‐W. Wu , T.‐T. Chang , C.‐C. Chang , J.‐W. Chen , Int. J. Mol. Sci. 2020, 21, 9245.33287461

[54] L. Xu , H. Zhang , Y. Wang , A. Yang , X. Dong , L. Gu , D. Liu , N. Ding , Y. Jiang , Lab Invest. 2022, 102, 25. 34725437 10.1038/s41374-021-00679-2 PMC8695379

[55] H. Cao , M. Sekiya , M. E. Ertunc , M. F. Burak , J. R. Mayers , A. White , K. Inouye , L. M. Rickey , B. C. Ercal , M. Furuhashi , G. Tuncman , G. S. Hotamisligil , Cell Metab. 2013, 17, 768. 23663740 10.1016/j.cmet.2013.04.012 PMC3755450

[56] M. F. Burak , K. E. Inouye , A. White , A. Lee , G. Tuncman , E. S. Calay , M. Sekiya , A. Tirosh , K. Eguchi , G. Birrane , D. Lightwood , L. Howells , G. Odede , H. Hailu , S. West , R. Garlish , H. Neale , C. Doyle , A. Moore , G. S. Hotamisligil , Sci. Transl. Med. 2015, 7, 319ra205. 10.1126/scitranslmed.aac6336 26702093

[57] X. Miao , Y. Wang , W. Wang , X. Lv , M. Wang , H. Yin , Mol. Cell. Endocrinol. 2015, 403, 1. 25596549 10.1016/j.mce.2014.12.017

[58] B. Liao , S. Yang , L. Geng , J. Zong , Z. Zhang , M. Jiang , X. Jiang , S. Li , A. Xu , J. Chang , R. L. C. Hoo , Br. J. Pharmacol. 2023, 181, 1238. 37949671 10.1111/bph.16282

[59] D. Beran , C. Abidha , A. Adler , C. de Beaufort , M. Lepeska , N. Levitt , E. Pfiester , J. H. Zafra‐Tanaka , E. A. Gale , Lancet Diabetes Endocrinol. 2023, 11, 78. 36623522 10.1016/S2213-8587(22)00384-9

[60] G. S. Hotamisligil , R. S. Johnson , R. J. Distel , R. Ellis , V. E. Papaioannou , B. M. Spiegelman , Science. 1996, 274, 1377. 8910278 10.1126/science.274.5291.1377

[61] G. E. Ryan , J. E. Harris , J. M. Richmond , Front. Immunol. 2021, 12, 652191. 34012438 10.3389/fimmu.2021.652191 PMC8128248

[62] Q. Zhao , T. Kim , J. Pang , W. Sun , X. Yang , J. Wang , Y. Song , H. Zhang , H. Sun , V. Rangan , S. Deshpande , H. Tang , M. E. Cvijic , R. Westhouse , T. Olah , J. Xie , M. Struthers , L. Salter‐Cid , J. Leukoc. Biol. 2017, 102, 1271. 28899907 10.1189/jlb.5A0717-302

[63] I. G. House , P. Savas , J. Lai , A. X. Y. Chen , A. J. Oliver , Z. L. Teo , K. L. Todd , M. A. Henderson , L. Giuffrida , E. V. Petley , K. Sek , S. Mardiana , T. N. Gide , C. Quek , R. A. Scolyer , G. V. Long , J. S. Wilmott , S. Loi , P. K. Darcy , P. A. Beavis , Clin. Cancer Res. 2020, 26, 487. 31636098 10.1158/1078-0432.CCR-19-1868

[64] J.‐B. Xia , G.‐H. Liu , Z.‐Y. Chen , C.‐Z. Mao , D.‐C. Zhou , H.‐Y. Wu , K.‐S. Park , H. Zhao , S.‐K. Kim , D.‐Q. Cai , X.‐F. Qi , Cytokine. 2016, 81, 63. 26891076 10.1016/j.cyto.2016.02.007

[65] R. Blanco‐Domínguez , H. de la Fuente , C. Rodríguez , L. Martín‐Aguado , R. Sánchez‐Díaz , R. Jiménez‐Alejandre , I. Rodríguez‐Arabaolaza , A. Curtabbi , M. M. García‐Guimaraes , A. Vera , F. Rivero , J. Cuesta , L. J. Jiménez‐Borreguero , A. Cecconi , A. Duran‐Cambra , M. Taurón , J. Alonso , H. Bueno , M. Villalba‐Orero , J. A. Enríquez , S. C. Robson , F. Alfonso , F. Sánchez‐Madrid , J. Martínez‐González , P. Martín , J. Clin. Invest. 2022, 132, e152418. 36066993 10.1172/JCI152418 PMC9621142

[66] A. Visperas , D. A. Vignali , J. Immunol. 2016, 197, 3762. 27815439 10.4049/jimmunol.1601118 PMC5119643

[67] G. J. Godoy , D. A. Paira , C. Olivera , M. L. Breser , L. R. Sanchez , R. D. Motrich , V. E. Rivero , Immunol. Lett. 2020, 223, 17. 32330480 10.1016/j.imlet.2020.04.006

[68] L. Shu , R. L. C. Hoo , X. Wu , Y. Pan , I. P. C. Lee , L. Y. Cheong , S. R. Bornstein , X. Rong , J. Guo , A. Xu , Nat. Commun. 2017, 8, 14147. 28128199 10.1038/ncomms14147 PMC5290165

[69] A. L. O'Kell , C. Wasserfall , B. Catchpole , L. J. Davison , R. S. Hess , J. A. Kushner , M. A. Atkinson , Diabetes. 2017, 66, 1443. 28533295 10.2337/db16-1551 PMC5440022

