# Supplementary material for: Islet‐Resident Memory T Cells Orchestrate the Immunopathogenesis of Type 1 Diabetes through the FABP4‐CXCL10 Axis
Source: Adv Sci (Weinh). 2024 Jun 17;11(30):2308461. doi: 10.1002/advs.202308461 (PMC11321687; doi:10.1002/advs.202308461)
Supplement: Supplementary file 1 — Supporting Information [file ADVS-11-2308461-s001.pdf]

ADVANCED SCIENCE
Open Access

Supporting Information

for *Adv. Sci.*, DOI 10.1002/advs.202308461

Islet-Resident Memory T Cells Orchestrate the Immunopathogenesis of Type 1 Diabetes through the FABP4-CXCL10 Axis

Xiaoping Wu, Lai Yee Cheong, Lufengzi Yuan, Leigang Jin, Zixuan Zhang, Yang Xiao, Zhiguang Zhou, Aimin Xu, Ruby LC Hoo and Lingling Shu**

Fig S1

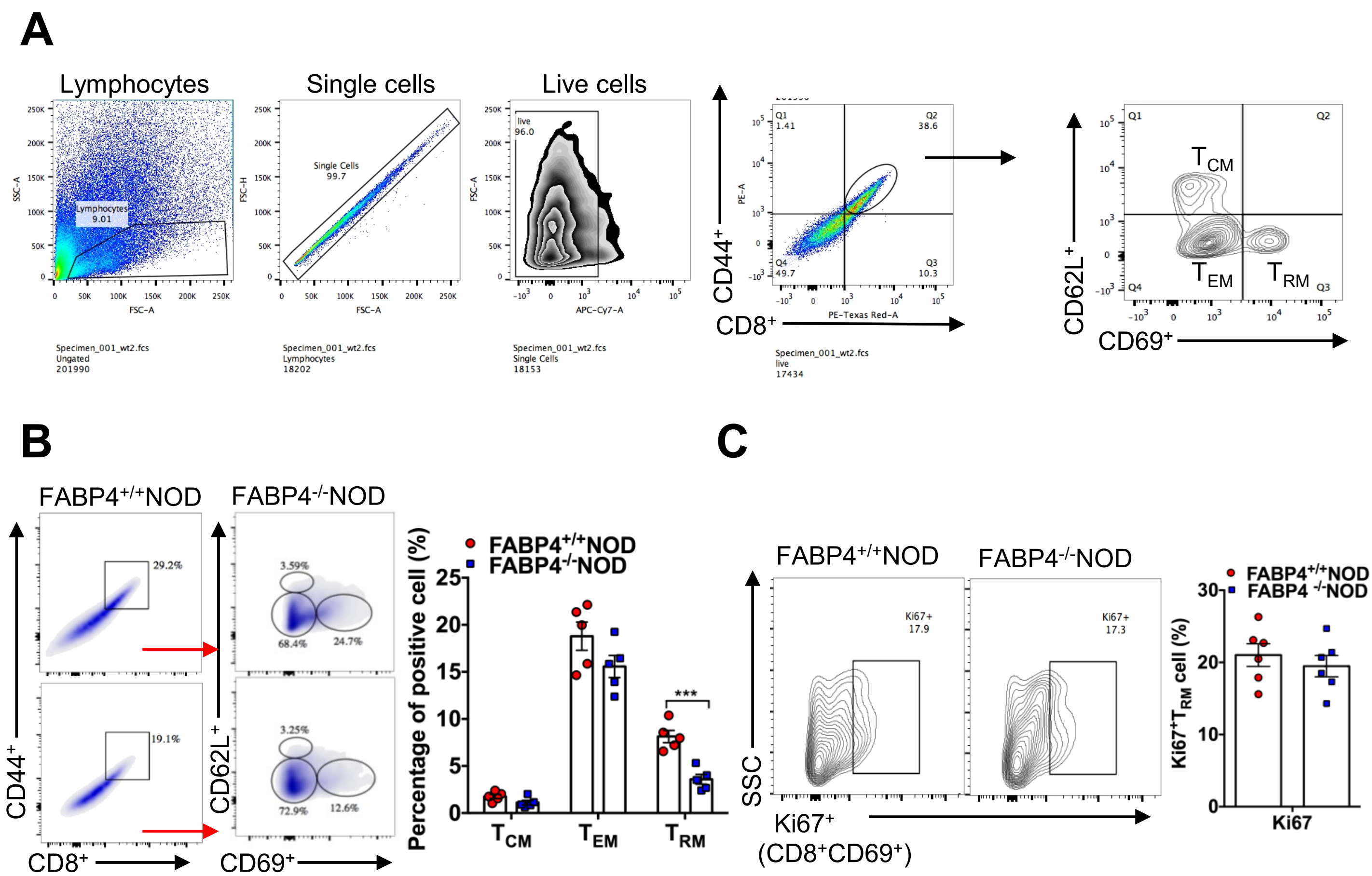

Fig S1. Involvement of FABP4 in the infiltration of adaptive immune cells and the survival of T_{RM} cells in the pancreas of NOD mice.

(A) Gating strategy of T_{CM} ($CD8^+CD44^+CD69^-CD62L^+$), T_{EM} ($CD8^+CD44^+CD69^-CD62L^-$) and T_{RM} ($CD8^+CD44^+CD69^+CD62L^-$) cells isolated from the pancreas of 10-week-old NOD mice.

(B) Representative FACS plots showing the percentage of T_{CM}, T_{EM}, and T_{RM} cells in the pancreas of 10-week-old $FABP4^{+/+}$NOD and $FABP4^{-/-}$NOD mice. The right panel is the percentage of respective cell types (n = 5).

(C) Representative FACS plots showing the Ki67 positive $FABP4^{+/+}$ T_{RM} and $FABP4^{-/-}$ T_{RM} cells isolated from the pancreas of NOD mice. The right panel is the percentage of Ki67 positive cells (n = 6).

Data are expressed as mean ± SD. *$p<0.05$, **$p<0.01$, ***$p<0.001$.

Fig S2

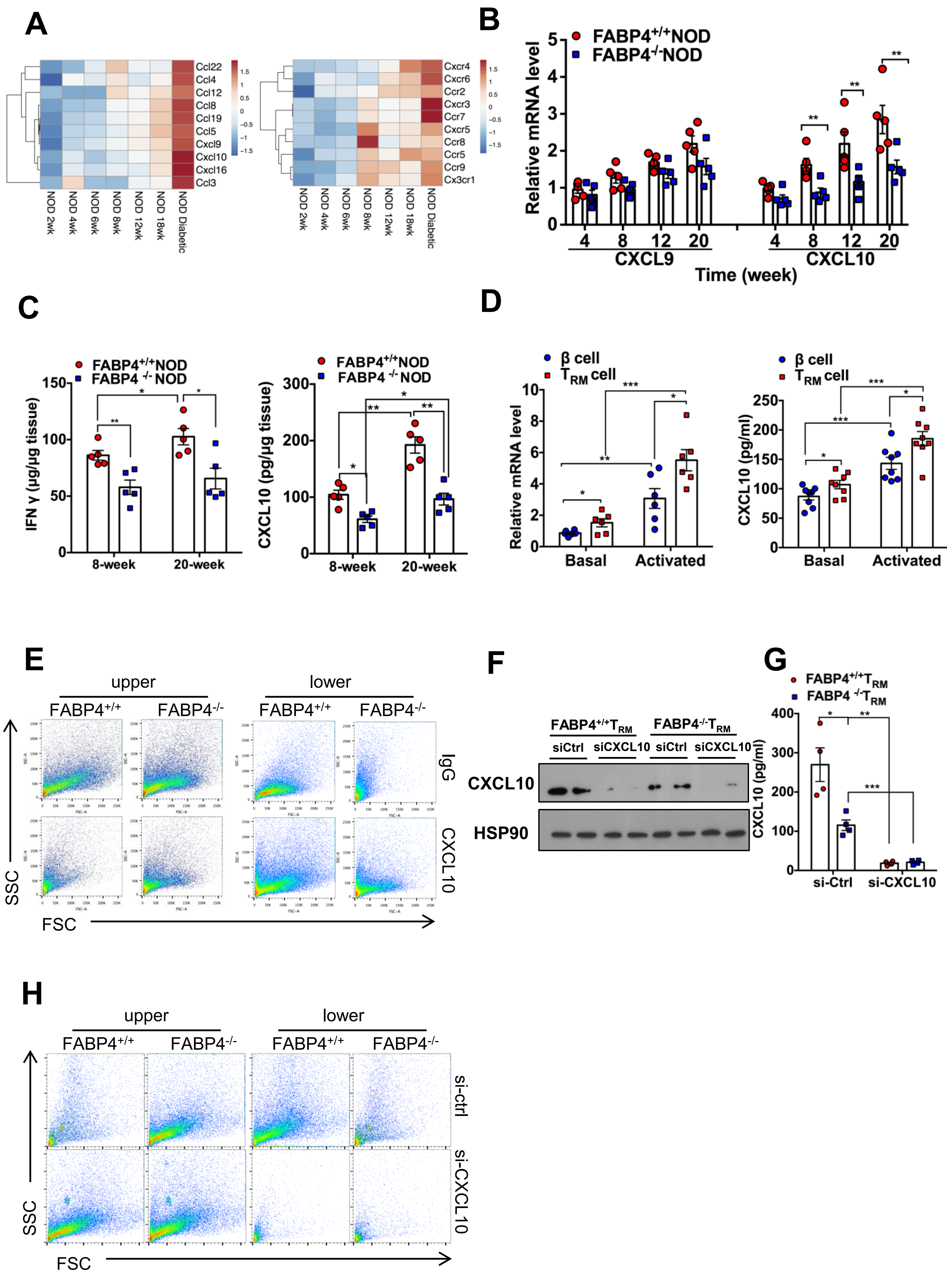

A
Ccl22
Ccl4
Ccl12
Ccl8
Ccl19
Ccl5
Cxcl9
Cxcl10
Cxcl16
Ccl3
Cxcr4
Cxcr6
Ccr2
Cxcr3
Ccr7
Cxcr5
Ccr8
Ccr5
Ccr9
Cx3cr1
NOD 2wk
NOD 4wk
NOD 6wk
NOD 8wk
NOD 12wk
NOD 18wk
NOD Diabetic
B
FABP4+/+NOD
FABP4-/-NOD
Relative mRNA level
CXCL9
CXCL10
Time (week)
C
IFN γ (µg/µg tissue)
CXCL10 (pg/µg tissue)
8-week
20-week
D
β cell
TRM cell
Relative mRNA level
CXCL10 (pg/ml)
Basal
Activated
E
upper
lower
FABP4+/+
FABP4-/-
IgG
CXCL10
SSC
FSC
F
FABP4+/+TRM
FABP4-/-TRM
siCtrl
siCXCL10
CXCL10
HSP90
G
FABP4+/+TRM
FABP4 -/-TRM
CXCL10 (pg/ml)
si-Ctrl
si-CXCL10
H
upper
lower
FABP4+/+
FABP4-/-
si-ctrl
si-CXCL10
SSC
FSC

Fig S2 FABP4 promotes CXCL10 expression whereby enhancing effector T cell recruitment

(A) Microarray data from the GEO database (GSE41203) showing the expression of chemokine (left) and chemokine receptors (right) in islets of NOD mice at different ages.

(B) The mRNA abundance of CXCL9 and CXCL10 in the pancreas of $FABP4^{+/+}$NOD and $FABP4^{-/-}$NOD mice at different ages (n = 5).

(C) The concentration of IFN γ (left) and CXCL10 (right) in pancreas lysate of 8-week-old and 20-week-old $FABP4^{+/+}$NOD and $FABP4^{-/-}$NOD mice (n = 5).

(D) The relative mRNA abundance of CXCL10 (left panel) and the concentration of CXCL10 in the culture medium (right panel) of primary beta and T_{RM} cells stimulated with $CD3^{+}CD28^{+}$ beads or vehicle (n = 6).

(E) Representative FACS plots showing the resident and migrated effector T cells cultured in a Trans-well plate with a conditioned medium of $FABP4^{+/+}$ T_{RM} cells or $FABP4^{-/-}$ T_{RM} cells subjected to the treatment of recombinant CXCL10 protein or IgG (served as a control).

(F) Representative immunoblots of CXCL10 and housekeeping control HSP90 in T_{RM} cells.

(G) The concentration of CXCL10 in the conditioned medium of $FABP4^{+/+}T_{RM}$ cells or $FABP4^{-/-}$ T_{RM} cells transfected with siControl (siCtrl) or siCXCL10 (n = 4).

(H) Representative FACS plots showing the resident and migrated effector T cells cultured in a Trans-well plate with the conditioned medium of $FABP4^{+/+}T_{RM}$ cells or $FABP4^{-/-}$ T_{RM} cells treated with siCXCL10 or siCtrl.

Data are expressed as mean ± SD. *p<0.05, **p<0.01, ***p<0.001.

Fig S3

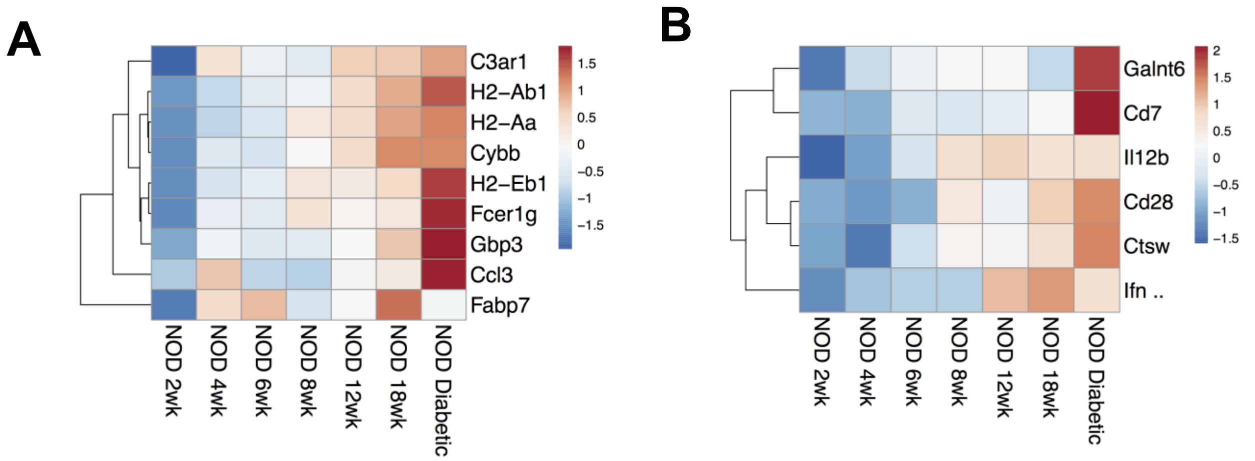

Fig S3. The timepoint of myeloid cell activation and T cell activation in the islet of NOD mice.

(A-B) Microarray data from the GEO database (GSE41203) shows the expression of genes related to (A) early myeloid cell activation and (B) T cell activation in islets of NOD mice at different ages as indicated.
